# Author Correction: Traceless native chemical ligation of lipid-modified peptide surfactants by mixed micelle formation

**DOI:** 10.1038/s41467-021-22508-2

**Published:** 2021-04-07

**Authors:** Shuaijiang Jin, Roberto J. Brea, Andrew K. Rudd, Stuart P. Moon, Matthew R. Pratt, Neal K. Devaraj

**Affiliations:** 1 Department of Chemistry and Biochemistry, University of California, San Diego, 9500 Gilman Drive, La Jolla, CA 92093 USA; 2 Department of Chemistry, University of Southern California, Los Angeles, CA 90089 USA

**Keywords:** Ubiquitins, Chemical tools, Peptides

Correction to: *Nature Communications* 10.1038/s41467-020-16595-w, published online 3 June 2020.

This article contains an error in Fig. 1b, in which the Dap(PhCn) amino acid was missing a methylene group between the nitrogen and the aromatic ring. The correct version of Fig. 1b is:

**Figure 1:**
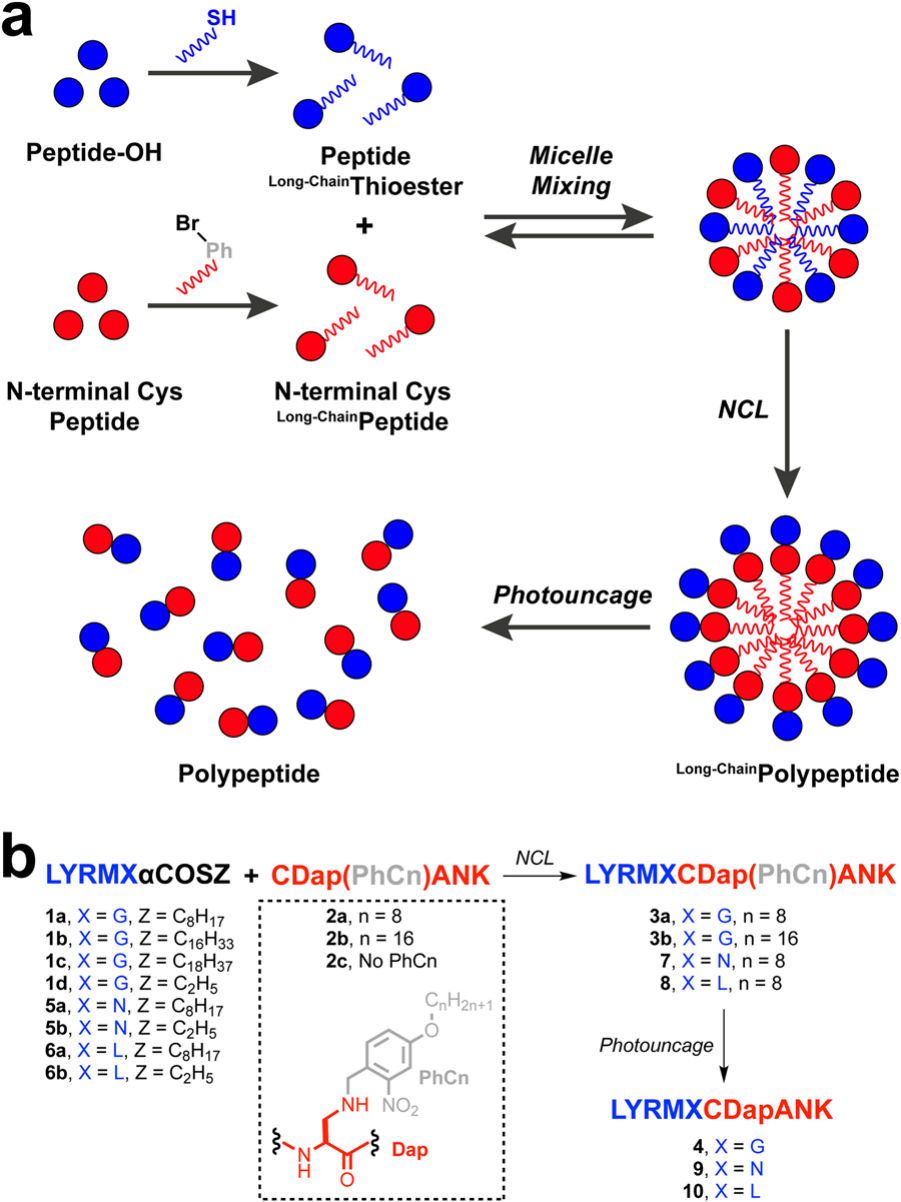
Fig. 1

The error has been corrected in the PDF and HTML versions of the article.

