# Supplementary material for: Traceless native chemical ligation of lipid-modified peptide surfactants by mixed micelle formation
Source: Nat Commun. 2020 Jun 3;11:2793. doi: 10.1038/s41467-020-16595-w (PMC7270136; doi:10.1038/s41467-020-16595-w)
Supplement: Supplementary file 1 — Supplementary Information [file 41467_2020_16595_MOESM1_ESM.pdf]

Supplementary Information

Traceless Native Chemical Ligation of Lipid-Modified Peptide Surfactants by Mixed Micelle Formation

Shuaijiang Jin[1], Roberto J. Brea[1], Andrew K. Rudd[1], Stuart P. Moon[2], Matthew R. Pratt[2], Neal K. Devaraj[1]*

[1]*Department of Chemistry and Biochemistry, University of California, San Diego, 9500 Gilman Drive*; *Natural Sciences Building, Room 3328, La Jolla, CA 92093, USA*

[2]*Department of Chemistry, University of Southern California, Los Angeles, CA 90089, USA*

Phone: (+1) 858 534 9539

E-mail: ndevaraj@uscd.edu

Supplementary Methods

General Considerations

Commercially available N_α-Fmoc-*L*-2,3-diaminopropionic acid (Fmoc-Dap-OH), Fmoc-Dap(Boc)-OH and 2-chlorotrityl chloride resin were obtained from Chem-Impex International, Inc. Fmoc-Ile-OH, Fmoc-Phe-OH, Fmoc-Leu-OH, Fmoc-His(Trt)-OH, Fmoc-Ser(tBu)-OH, Fmoc-Gly-OH, Fmoc-Met-OH, Fmoc-Arg(Pbf)-OH, Fmoc-Ala-OH, Fmoc-Lys(Boc)-OH, Fmoc-Asn(Trt)-OH, Fmoc-Tyr(tBu)-OH, Fmoc-Val-OH, Fmoc-Glu(*t*Bu)-OH, Boc-Cys(Trt)-OH, Boc-Leu-OH, 4-Methyl-3-nitrophenol, 1-bromohexadecane, Fmoc-Asn(Trt)-OH, Fmoc-Ser(*t*Bu)-OH, 1-bromooctane, *N*-bromosuccinimide (NBS), azobisisobutyronitrile (AIBN), *N,N,N',N'*-tetramethyl-*O*-(1*H*-benzotriazol-1-yl)uronium hexafluorophosphate (HBTU), *N,N*-diisopropylethylamine (DIEA), 1-hydroxybenzotriazole monohydrate (HOBt·H_2O), trifluoroacetic acid (TFA), phenol, thioanisole, 1,2-ethanedithiol (EDT), benzotriazole-1-yl-oxy-tris-pyrrolidino-phosphonium hexafluorophosphate (PyBOP), 1,1,1,3,3,3-hexafluoro-2-propanol (HFIP), palladium-tetrakis(triphenylphosphine), phenylsilane, guanidinium chloride (Gn·HCl), 1-octanethiol, 1-hexadecanethiol, 2-methylpropane-2-thiol, ethanethiol, sodium diethyldithiocarbamate trihydrate, potassium carbonate (K_2CO_3), dichloromethane (DCM or CH_2Cl_2), acetonitrile (CH_3CN), diethyl ether (Et_2O) and *N,N*-dimethylformamide (DMF) were obtained from Sigma-Aldrich. Bond-Breaker™ TCEP Solution (neutral pH) was obtained from Thermo Fisher. 2,2'-Azobis[2-(2-imidazolin-2-yl)propane] dihydrochloride (VA-044) was obtained from TCI America. Deuterated chloroform ($CDCl_3$) was obtained from Cambridge Isotope Laboratories. All reagents obtained from commercial suppliers were used without further purification. Analytical thin-layer chromatography was performed on E. Merck silica gel 60 F_{254} plates. Silica gel flash chromatography was performed using E. Merck silica gel (type 60SDS, 230-400 mesh). Solvent mixtures for chromatography are reported as v/v ratios. HPLC analysis was carried out on an Eclipse Plus C8 analytical column with *Phase A*/*Phase B* gradients [*Phase A*: H_2O with 0.1% formic acid; *Phase B*: MeOH with 0.1% formic acid]. HPLC purification was carried out on Zorbax SB-C18 semipreparative column with *Phase A*/*Phase B* gradients [*Phase A*: H_2O with 0.1% formic acid; *Phase B*: MeOH with 0.1% formic acid]. Proton nuclear magnetic resonance (^{1}H NMR) spectra were recorded on a VarianVX-500 MHz spectrometer, and were referenced relative to residual proton resonances in $CDCl_3$ (at 7.24 ppm). Chemical shifts were reported in parts per million (ppm, δ) relative to tetramethylsilane (at 0.00 ppm). ^{1}H NMR splitting patterns are assigned as singlet (s), doublet (d), triplet (t), quartet (q) or pentuplet (p). All first-order splitting patterns were designated on the basis of the appearance of the multiplet. Splitting patterns that could not be readily interpreted are designated as multiplet (m) or broad (br). Carbon nuclear magnetic resonance (^{13}C NMR) spectra were recorded on a Varian VX-500 MHz spectrometer, and were referenced relative to residual proton resonances in $CDCl_3$ (at 77.23 ppm). Electrospray Ionization-Time of Flight (ESI-TOF) spectra were obtained on an Agilent 6230 Accurate-Mass TOF mass spectrometer. Photocleavage reaction experiments were carried out on a Rayonet photochemical reactor (The Southern New England Ultraviolet Co., USA) equipped with UV lamps (365 nm). Wilmad quartz (CFQ) EPR tubes (o.d. = 5 mm, L = 100 mm) were obtained from Sigma-Aldrich. Dynamic light

scattering (DLS) experiments were carried out on a Malvern Instruments Zetasizer Nano systems DLS instrument (Malvern Inc., Worcestershire, UK).

Synthesis of Fmoc-Dap(PhC8)-OH (**S3b**)

Peptide **S3b** was synthesized according to Supplementary Fig. 47.

1-Methyl-2-nitro-4-(octyloxy)benzene (S1b). A solution of 4-methyl-3-nitrophenol (5.00 g, 32.68 mmol) in CH_3CN (200 mL) was treated with K_2CO_3 (8.13 g, 58.82 mmol) and 1-bromooctane (6.77 mL, 39.21 mmol). Then the reaction mixture was stirred for 24 h at reflux (~ 82 °C; with a condenser). Afterwards, the resulting suspension was filtered to remove the potassium carbonate. The solvent was removed *in vacuo* and the crude was purified by flash chromatography (0-10% EtOAc in hexanes) to afford **S1b** as a yellowish solid (8.24 g, 95%). ^{1}H NMR: (500 MHz, $CDCl_3$, δ): 7.50 (d, J = 2.7 Hz, 1H), 7.22 (d, J = 8.5 Hz, 1H), 7.05 (dd, J_1 = 8.4 Hz, J_2 = 2.7 Hz, 1H), 3.98 (t, J = 6.5 Hz, 2H), 2.52 (s, 3H), 1.84-1.75 (m, 2H), 1.54-1.44 (m, 2H), 1.44-1.22 (m, 10H), 0.89 (t, J = 6.8 Hz, 3H) (Supplementary Fig. 43). ^{13}C NMR (126 MHz, $CDCl_3$, δ): 157.60, 149.31, 133.37, 125.29, 120.43, 109.60, 68.63, 31.80, 29.31, 29.23, 29.04, 25.96, 22.67, 19.79, 14.12 (Supplementary Fig. 43).

1-(Bromomethyl)-2-nitro-4-(octyloxy)benzene (S2b). A solution of **S1b** (2.00 g, 7.54 mmol) in CCl_4 (15 mL) was placed in a flame-dried 2-neck round bottom flask with a condenser and then successively treated with recrystallized NBS (1.48 g, 8.29 mmol) and AIBN (123.16 mg, 0.75 mmol). The resulting suspension was irradiated with white light (250 W Philips tungsten bulb) under reflux for 10 h. After this period of time, the light was removed, and the reaction was cooled to rt. The obtained succinimide was filtered off and washed with CH_2Cl_2 (15 mL). The filtrate was then added to a separatory funnel and washed with $NaHCO_3$ (sat.) (10 mL), H_2O (10 mL) and NaCl (sat.) (10 mL). The organic phase was dried over anhydrous Na_2SO_4 and concentrated *in vacuo*. The resulting crude material was then purified by flash chromatography (0-10% EtOAc in hexanes) to afford **S2b** as an off-white solid (1.37 g, 53%). ^{1}H NMR (500 MHz, $CDCl_3$, δ): 7.54 (d, J = 2.7 Hz, 1H), 7.44 (dd, J_1 = 8.6 Hz, J_2 = 2.4 Hz, 1H), 7.11 (dt, J_1 = 8.6 Hz, J_2 = 2.7 Hz, 1H), 4.80 (s, 1H), 4.02 (t, J = 6.7 Hz, 1H), 1.83-1.78 (m, 2H), 1.54-1.41 (m, 2H), 1.44-1.22 (m, 10.4 Hz, 10H), 0.89 (t, J = 5.7 Hz, 2H) (Supplementary Fig. 44). ^{13}C NMR (126 MHz, $CDCl_3$, δ): 159.61, 148.45, 133.49, 124.37, 120.24, 110.82, 68.86, 31.74, 29.31, 29.22, 29.16, 28.87, 25.86, 22.61, 14.08 (Supplementary Fig. 44).

(*R*)-2-((((9*H*-fluoren-9-yl)methoxy)carbonyl)amino)-3-((2-nitro-4-(octyloxy)benzyl)-amino)propanoic acid (S3b). To a solution of **S2b** (1.00 g, 2.92 mmol) in MeOH:CH_2Cl_2 (4:1; 45 ml) was added Fmoc-Dap-OH (1.14 g, 3.50 mmol), and the mixture was stirred at rt for 5 min. The resulting suspension was then treated with DIEA (1.52 mL, 8.76 mmol), which caused the suspension to gradually clear. The reaction mixture was stirred at rt for 20 h shielded from light. Afterwards, the solvent was removed *in vacuo*, while maintaining the temperature of water bath around 20 °C to avoid potential decomposition and/or side reactions. Then, the obtained crude was purified by flash chromatography (0-5% MeOH in CH_2Cl_2), affording **S3b** as a yellowish foam (1.24 g, 72%). ^{1}H NMR (500 MHz, $CDCl_3$, δ): 7.64 (d, J = 7.6 Hz, 2H), 7.48 (q, J_1 = 9.4 Hz, J_2 = 6.3 Hz, 3H), 7.28 (td, J_1 = 7.4 Hz, J_2 = 3.7 Hz, 2H), 7.23-7.16 (m, 3H), 7.07-6.89 (m, 1H), 6.48 (br, 1H), 4.48-3.39 (m, 1H), 4.35-4.27 (m, 2H), 4.23 (t, J = 8.9 Hz, 1H), 4.14 (t, J = 8.9 Hz, 1H), 4.06 (t, J = 7.4 Hz, 1H), 3.77 (t, J = 6.7 Hz, 2H), 3.57-3.39 (m, 2H),

1.65-1.56 (m, 2H), 1.32-1.26 (m, 2H), 1.26-1.12 (m, 10H), 0.81 (t, J = 6.2 Hz, 3H). (Supplementary Fig. 45). ^{13}C NMR (126 MHz, $CDCl_3$, δ): 172.40, 160.82, 156.69, 149.38, 143.72, 143.67, 141.15, 135.47, 127.70, 127.14, 125.30, 125.26, 120.58, 119.87, 116.96, 111.61, 68.98, 67.64, 51.75, 49.77, 49.29, 46.85, 31.94, 31.78, 29.72, 29.27, 29.18, 28.90, 28.83, 25.81, 22.66, 14.13 (Supplementary Fig.45). **HRMS (ESI-TOF)** calculated for $[C_{33}H_{38}N_3O_7]^-$ ($[M-H]^-$) 588.2715, found 588.2711.

Synthesis of CDap(PhCn)ANK peptides

CDap(PhC8)ANK (2a). Using the general procedure I, 12.56 mg of **2a** was obtained (white solid, 16%). Analytical HPLC: t_R = 2.30 min (50 to 95% *Phase B* over 1 min, then 95% *Phase B* for 5 min, Eclipse Plus C8 analytical column). MS (ESI) $[C_{34}H_{57}N_9O_{10}S]$ calculated: 784.4 $[M+H]^+$, 392.7 $[M+2H]^{2+}$; found: 784.3 $[M+H]^+$, 392.8 $[M+2H]^{2+}$ (Supplementary Fig. 2).

CDapANK (2c). Using the general procedure I, 16.60 mg of **2c** was obtained (white solid, 32%). Analytical HPLC: t_R = 0.68 min (50 to 95% *Phase B* over 1 min, then 95% *Phase B* for 5 min, Eclipse Plus C8 analytical column). MS (ESI) $[C_{19}H_{36}N_8O_7S]$ calculated 521.2 $[M+H]^+$, 261.1 $[M+2H]^{2+}$; found 522.3 $[M+H]^+$, 261.2 $[M+2H]^{2+}$ (Supplementary Fig. 3).

Synthesis of LYRMX-Z peptides

LYRMG-αCOSC16 (1b). Using the general procedure II, 4.88 mg of **1b** were obtained (white solid, 58% for two steps from 10.00 mg of Boc-Leu-Tyr(^{t}Bu)-Arg(Pbf)-Met-Gly-OH). Analytical HPLC: t_R = 2.93 min (50 to 95% *Phase B* over 1 min, then 95% *Phase B* for 5 min, Eclipse Plus C8 analytical column). MS (ESI) $[C_{44}H_{78}N_8O_6S_2]$ calculated 879.5 $[M+H]^+$, 440.3 $[M+2H]^{2+}$; found 879.5 $[M+H]^+$, 440.4 $[M+2H]^{2+}$ (Supplementary Fig. 6).

LYRMG-αCOSC18 (1c). Using the general procedure II, 4.77 mg of **1c** were obtained (white solid, 55% for two steps from 10.00 mg of Boc-Leu-Tyr(^{t}Bu)-Arg(Pbf)-Met-Gly-OH). Analytical HPLC: t_R = 2.6 min (50 to 95% *Phase B* over 8.5 min, then 95% *Phase B* for 7 min, Eclipse Plus C8 analytical column). MS (ESI) $[C_{46}H_{82}N_8O_6S_2]$ calculated 906.6 $[M+H]^+$, 454.3 $[M+2H]^{2+}$; found 907.5 $[M+H]^+$, 454.4 $[M+2H]^{2+}$ (Supplementary Fig. 7).

LYRMG-αCOSC2 (1d). Using the general procedure II, 4.70 mg of **1d** were obtained (white solid, 72% for two steps from 10.00 mg of Boc-Leu-Tyr(^{t}Bu)-Arg(Pbf)-Met-Gly-OH). Analytical HPLC: t_R = 0.47 min (50 to 95% *Phase B* over 1 min, then 95% *Phase B* for 5 min, Eclipse Plus C8 analytical column). MS (ESI) $[C_{30}H_{50}N_8O_6S_2]$ calculated 683.3 $[M+H]^+$, 342.2 $[M+2H]^{2+}$; found 683.2 $[M+H]^+$, 342.2 $[M+2H]^{2+}$ (Supplementary Fig. 8).

LYRMN-αCOSC8 (5a). Using the general procedure II, 2.85 mg of **5a** were obtained [white solid, 46% for two steps from 10.00 mg of Boc-Leu-Tyr(^{t}Bu)-Arg(Pbf)-Met-Asn(Trt)-OH]. Analytical HPLC: t_R = 4.58 min (20 to 95% *Phase B* over 5 min, then 95% *Phase B* for 7 min, Eclipse Plus C8 analytical column). MS (ESI) $[C_{38}H_{65}N_9O_7S_2]$ calculated 824.4 $[M+H]^+$, 412.7 $[M+2H]^{2+}$; found 824.3 $[M+H]^+$, 412.8 $[M+2H]^{2+}$ (Supplementary Fig. 9).

LYRMN-αCOSC2 (5b). Using the general procedure II, 4.90 mg of **5b** were obtained [white solid, 59% for two steps from 10.00 mg of Boc-Leu-Tyr(^{t}Bu)-Arg(Pbf)-Met-

Asn(Trt)-OH]. Analytical HPLC: t_R = 0.99 min (50 to 95% *Phase B* over 1 min, then 95% *Phase B* for 5 min, Eclipse Plus C8 analytical column)MS (ESI) [$C_{32}H_{53}N_9O_7S_2$] calculated 740.4 $[M+H]^+$, 370.7 $[M+2H]^{2+}$; found 740.2 $[M+H]^+$, 370.8 $[M+2H]^{2+}$ (Supplementary Fig. 10).

LYRML-αCOSC8 (6a). Using the general procedure II, 2.45 mg of **6a** were obtained [white solid, 33% for two steps from 10.00 mg of Boc-Leu-Tyr(^{t}Bu)-Arg(Pbf)-Met-Leu-OH]. Analytical HPLC: t_R = 5.10 min (20 to 95% *Phase B* over 5 min, then 95% *Phase B* for 7 min, Eclipse Plus C8 analytical column). MS (ESI) [$C_{40}H_{70}N_8O_6S_2$] calculated 823.5 $[M+H]^+$, 413.3 $[M+2H]^{2+}$; found 823.4 $[M+H]^+$, 412.4 $[M+2H]^{2+}$ (Supplementary Fig. 11).

LYRML-αCOSC2 (6b). Using the general procedure II, 3.50 mg of **6b** were obtained [white solid, 52% for two steps from 10.00 mg of Boc-Leu-Tyr(^{t}Bu)-Arg(Pbf)-Met-Leu-OH]. Analytical HPLC: t_R = 1.24 min (50 to 95% Phase B over 1 min, then 95% Phase B for 5 min, Eclipse Plus C8 analytical column). MS (ESI) [$C_{34}H_{58}N_8O_6S_2$] calculated 739.4 $[M+H]^+$, 370.2 $[M+2H]^{2+}$; found 739.2 $[M+H]^+$, 370.2 $[M+2H]^{2+}$ (Supplementary Fig. 12).

Native chemical ligation

All the NCL reactions for synthesizing decapeptides were performed following the general procedure III.

LYRMGCDap(PhC8)ANK (3a). Using the general procedure III for NCL, **3a** was synthesized. Analytical HPLC: t_R = 12.64 min (0% *Phase B* for 1 min, then 0 to 95% *Phase B* over 19 min, then 95% *Phase B* for 2 min, Eclipse Plus C8 analytical column). MS (ESI) [$C_{62}H_{101}N_{17}O_{16}S_2$] calculated 702.9 $[M+2H]^{2+}$, 768.9 $[M+3H]^{3+}$; found 703.3 $[M+2H]^{2+}$, 469.0 $[M+3H]^{3+}$ (Supplementary Fig. 14).

LYRMNCDap(PhC16)ANK (7). Using the general procedure III for NCL, **7** was synthesized. Analytical HPLC: t_R = 13.10 min (5% *Phase B* for 1 min, then 5 to 95% *Phase B* over 14 min, then 95% *Phase B* for 2 min, Eclipse Plus C8 analytical column). MS (ESI) [$C_{72}H_{120}N_{18}O_{17}S_2$] calculated 787.5 $[M+2H]^{2+}$, 525.3 $[M+3H]^{3+}$; found 787.9 $[M+2H]^{2+}$, 525.3 $[M+3H]^{3+}$. Note: Construction of Asn-Cys junction via NCL reaction may generate β-linked isopeptidic product.[1] However, via exhaustive analysis of the obtained HPLC-MS traces, no peak (other than the desired ligation product peak) corresponding to the isopeptidic product was identified (Supplementary Fig. 15).

LYRMLCDap(PhC16)ANK (8). Using the general procedure III for NCL, **8** was synthesized. Analytical HPLC: t_R = 20.58 min (0% *Phase B* for 1 min, then 0 to 95% *Phase B* over 22 min, then 95% *Phase B* for 2 min, Eclipse Plus C8 analytical column). MS (ESI) [$C_{74}H_{125}N_{17}O_{16}S_2$] calculated 787.0 $[M+2H]^{2+}$, 525.0 $[M+3H]^{3+}$; found 787.0 $[M+2H]^{2+}$, 525.1 $[M+3H]^{3+}$ (Supplementary Fig. 16).

Photouncaging reactions

All the photouncaging reactions were performed following the general procedure IV.

Preparation of LYRMNCDapANK (9). Using the general procedure IV for photouncaging reaction, **9** was synthesized from LYRMNCDap(PhC16)ANK (**7**). Analytical HPLC: t_R = 3.71 min (5% *Phase B* for 1 min, then 5 to 95% *Phase B* over 14 min, then 95% *Phase B* for 2 min, Eclipse Plus C8 analytical column). MS (ESI) [$C_{49}H_{83}N_{17}O_{14}S_2$] calculated 599.8 $[M+2H]^{2+}$, 400.2 $[M+3H]^{3+}$, 300.4 $[M+4H]^{4+}$; found 600 $[M+2H]^{2+}$, 400.3 $[M+3H]^{3+}$, 300.6 $[M+4H]^{4+}$ (Supplementary Fig. 18).

Preparation of LYRMLCDapANK (10). Using the general procedure IV for photouncaging reaction, **10** was synthesized from LYRMLCDap(PhC16)ANK (**8**). Analytical HPLC: t_R = 5.75 min (0% *Phase B* for 1 min, then 0 to 95% *Phase B* over 22 min, then 95% *Phase B* for 2 min, Eclipse Plus C8 analytical column). MS (ESI) [$C_{51}H_{88}N_{16}O_{13}S_2$] calculated 599.3 $[M+2H]^{2+}$, 399.9 $[M+3H]^{3+}$, 300.2 $[M+4H]^{4+}$; found 599.2 $[M+2H]^{2+}$, 399.9 $[M+3H]^{3+}$, 300 $[M+4H]^{4+}$ (Supplementary Fig. 19).

Synthesis of LYRMGCKANK (**S6**)

Preparation of CK(PhC16)ANK (S4). Following the same strategy (Supplementary Fig. 53) than for the preparation of CK(PhC16)KFGKAFVGEIMNS (**12**), CK(PhC16)ANK (**S4**) was prepared via sequential SPPS, Alloc deprotection, and photocaging-group substitution. The sample was dissolved in MeOH and purified by HPLC using a Zorbax SB-C18 semipreparative column [50 to 95% *Phase B* for 10 min, then 95% *Phase B* for 8 min], obtaining CK(PhC16)ANK (**S4**) as a white solid. Analytical HPLC: t_R = 4.26 min (50 to 95% *Phase B* over 3 min, then 95% *Phase B* for 5 min, Eclipse Plus C8 analytical column). MS (ESI) [$C_{45}H_{79}N_9O_{10}S$] calculated: 469.8 $[M+2H]^{2+}$; found 469.8 $[M+2H]^{2+}$ (Supplementary Fig. 20).

LYRMGCK(PhC16)ANK (S5). As shown in Supplementary Fig. 52, CK(PhC16)ANK (**S4**) and LYRMG-αCOSC8 (**1a**) were subjected to the general procedure III for NCL, and LYRMGCK(PhC16)ANK (**S5**) was synthesized. Analytical HPLC: t_R = 13.00 min (0% *Phase B* for 1 min, then 0 to 95% *Phase B* over 14 min, then 95% *Phase B* for 2 min, Eclipse Plus C8 analytical column). MS (ESI) [$C_{73}H_{123}N_{17}O_{16}S_2$] calculated 779.9 $[M+2H]^{2+}$, 520.3 $[M+3H]^{3+}$, 390.5 $[M+4H]^{4+}$; found 780.0 $[M+2H]^{2+}$, 520.5 $[M+3H]^{3+}$,390.6 $[M+4H]^{4+}$ (Supplementary Fig. 21).

Preparation of LYRMGCKANK (S6). Using the general procedure IV for photouncaging reaction, LYRMGCKANK (**S6**) was synthesized from LYRMGCK(PhC16)ANK **(S5)**. Analytical HPLC: t_R = 4.40 min (5% *Phase B* for 1 min, then 5 to 95% *Phase B* over 14 min, then 95% *Phase B* for 2 min, Eclipse Plus C8 analytical column). MS (ESI) [$C_{50}H_{86}N_{16}O_{13}S_2$] calculated 592.3 $[M+2H]^{2+}$, 603.3 $[M+H+Na]^{2+}$, 395.2 $[M+3H]^{3+}$; found 592.2 $[M+2H]^{2+}$, 603.1 $[M+H+Na]^{2+}$, 395.3 $[M+3H]^{3+}$ (Supplementary Fig. 22).

Control reaction for the synthesis of Magainin 2

The lyophilized peptides GIGKFLHS-αCOSC2 (**S8**, 1 mM) and CK(PhC16)KFGKAFVGEIMNS (**12**, 1 mM) were subjected to the same NCL condition (same ligation buffer with 2 mM SDS and temperature) used for the synthesis of Magainin 2 in *Methods*. After 24 h, no ligation product GIGKFLHSCK(PhC16)KFGKAFVGEIMNS (**13**) was observed (Supplementary Fig. 33).

Synthesis of LYRMGCADap(PhC16)NK (**S9**)

Preparation of CADap(PhC16)NK (S10). Using the general procedure I, CADap(PhC16)NK (**S10**) was prepared via successive SPPS and TFA cocktail deprotection. Then, the diethyl ether precipitated crude product was dissolved in MeOH and purified by HPLC using a Zorbax SB-C18 semipreparative column [50 to 95% *Phase B* for 10 min, then 95% *Phase B* for 8 min], obtaining CADap(PhC16)NK (**S10**) as a white solid. Analytical HPLC: t_R = 2.52 min (50 to 95% *Phase B* over 1 min, then 95% *Phase B*

for 5 min, Eclipse Plus C8 analytical column). MS (ESI) [$C_{42}H_{73}N_9O_{10}S$] calculated: 448.8 $[M+2H]^{2+}$; found 448.8 $[M+2H]^{2+}$ (Supplementary Fig. 34).

LYRMGCADap(PhC16)NK (S9). As shown in Supplementary Fig. 54, CADap(PhC16)NK (**S10**) and LYRMG-αCOSC8 (**1a**) were subjected to the general procedure III for NCL, and LYRMGCADap(PhC16)NK (**S9**) was synthesized. The reaction solution was gently shaken at room temperature. Aliquots were taken at various time intervals and analyzed by analytical HPLC-MS. Analytical HPLC: t_R = 13.49 min (0% *Phase B* for 1 min, then 0 to 95% *Phase B* over 14 min, then 95% *Phase B* for 2 min, Eclipse Plus C8 analytical column). MS (ESI) [$C_{70}H_{117}N_{17}O_{16}S_2$] calculated: 758.9 $[M+2H]^{2+}$, 506.3 $[M+3H]^{3+}$; found 759.4 $[M+2H]^{2+}$, 506.5 $[M+3H]^{3+}$. Supplementary Fig. 35 shows the comparison of the kinetics of NCL reaction of the peptide thioesters LYRMG-αCOSC8 (**1a**, 1 mM) with the cysteine-based peptide CDap(PhC16)ANK (**2b**) (1 mM, blue line) and CADap(PhC16)NK (**S10**) (1 mM, red line).

Comparison to NCL with MPAA additives

Preparation of LYRMGCDapANK (4). The reaction conditions for the NCL with MPAA additives are based on the standard reported method.[1] The lyophilized model peptides **1d** (0.2 μmol, 1.0 equiv.) and **2c** (0.2 μmol, 1.0 equiv.) were dissolved in separate vials using freshly degassed ligation buffer (200 μL for each vial respectively. 6 M guanidine hydrochloride, 200 mM Na_2HPO_4, 10 mM TCEP hydrochloride, and 20 mM MPAA. The pH was adjusted to 7.0). After sonication of both mixtures for 3 min, the solutions from both vials were combined (final concentration of each peptide: 500 μM). The resulting solution was gently shaken at rt. Aliquots were taken at various time intervals and analyzed by analytical HPLC-MS. Analytical HPLC: t_R = 4.90 min (0% *Phase B* for 1 min, then 0 to 95% *Phase B* over 14 min, then 95% *Phase B* for 2 min, Eclipse Plus C8 analytical column). MS (ESI) [$C_{47}H_{80}N_{16}O_{13}S_2$] calculated: 571.3 $[M+2H]^{2+}$, 381.2 $[M+3H]^{3+}$; found 571.3 $[M+2H]^{2+}$, 381.4 $[M+3H]^{3+}$.

Supplementary Fig. 36 shows the comparison of the kinetics of NCL reactions at 500 μM. Blue line: Lipid-templated NCL reaction between LYRMG-αCOSC8 (**1a**, 500 μM) and CDap(PhC16)ANK (**2b**, 500 μM). Red line: MPAA-catalyzed NCL reaction between LYRMG-αCOSC2 (**1d**, 500 μM) and CDapANK (**2c**, 500 μM).

Synthesis of LYRMGCK(PhC16)ANC (**S11**)

Preparation of CK(PhC16)ANC (S12). Following the same strategy Supplementary Fig. 53) than for the preparation of CK(PhC16)KFGKAFVGEIMNS (**12**), CK(PhC16)ANC (**S12**) was prepared via SPPS, Alloc deprotection, and photocaging-group substitution in sequence. The sample was dissolved in MeOH and purified by HPLC using a Zorbax SB-C18 semipreparative column [50 to 95% *Phase B* for 10 min, then 95% *Phase B* for 8 min], obtaining CK(PhC16)ANC (**S12**) as a white solid. Analytical HPLC: t_R = 5.62 min (50 to 95% *Phase B* over 3 min, then 95% *Phase B* for 5 min, Eclipse Plus C8 analytical column). MS (ESI) [$C_{42}H_{72}N_8O_{10}S_2$] calculated: 457.3 $[M+2H]^{2+}$; found 457.4 $[M+2H]^{2+}$ (Supplementary Fig. 37).

LYRMGCK(PhC16)ANC (S11). As shown in Supplementary Fig. 56, CK(PhC16)ANC (**S10**) and LYRMG-αCOSC8 (**1a**) were subjected to the general procedure III for NCL (except 20 mM TCEP was applied in this case), and LYRMGCK(PhC16)ANC (**S11**) was synthesized. The reaction solution was gently shaken

at room temperature. Aliquots were taken at various time intervals and analyzed by analytical HPLC-MS. Analytical HPLC: t_R = 14.05 min (0% *Phase B* for 1 min, then 0 to 95% *Phase B* over 14 min, then 95% *Phase B* for 2 min, Eclipse Plus C8 analytical column). MS (ESI) [$C_{70}H_{116}N_{16}O_{16}S_3$] calculated: 767.4 $[M+2H]^{2+}$, 511.9 $[M+3H]^{3+}$; found 767.5 $[M+2H]^{2+}$, 512.0 $[M+3H]^{3+}$. Supplementary Fig. 38 shows the kinetics of NCL reaction between the peptide thioesters LYRMG-αCOSC8 (**1a**) (1 mM) with the cysteine-based peptide CK(PhC16)ANC (**S12**). Supplementary Fig. 39 corresponds to the HPLC traces and MS-MS analysis spectrum of the product LYRMGCK(PhC16)ANC (**S11**).

Control reaction for the derivatization of Ubiquitin

Preparation of CKANK (S13). Using the general procedure I, 21.90 mg of **S13** was obtained (white solid, 39%). Analytical HPLC: t_R = 1.17 min (50 to 95% Phase B over 3 min, then 95% Phase B for 7 min, Eclipse Plus C8 analytical column). MS (ESI) [$C_{22}H_{42}N_8O_7S$] calculated 563.3 $[M+H]^+$, 282.2 $[M+2H]^{2+}$; found 563.4 $[M+H]^+$, 282.2 $[M+2H]^{2+}$ (Supplementary Fig. 60).

Control reaction for the derivatization of Ubiquitin. The lyophilized peptides Ubi-αCOSC8 (**16**, 0.5 mM) and CKANK (**S13**, 2 mM) were subjected to the same NCL condition (same ligation buffer with 10 mM SDS and temperature) used for the derivatization of Ubiquitin in *Methods*. After 5 h, minimal amount of ligation product Ubi-CKANK (**S14**, t_R = 11.20 min) was generated (6% conversion, Supplementary Fig. 61) and verified by mass spectrometry (ESI-TOF, Supplementary Fig. 62). The major peak (t_R = 12.90 min) of the HPLC spectrum was also verified by mass spectrometry (ESI-TOF) to be the remained starting material Ubi-αCOSC8 (**16**).

Supplementary Figures

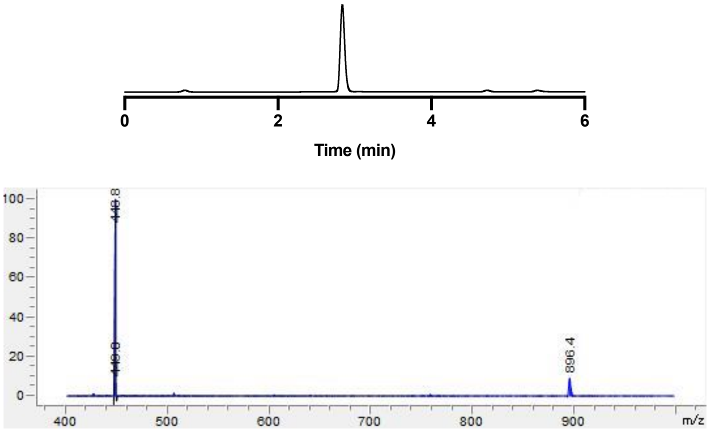

Supplementary Figure 1. HPLC trace and MS (ESI) of purified CDap(PhC16)ANK (**2b**).

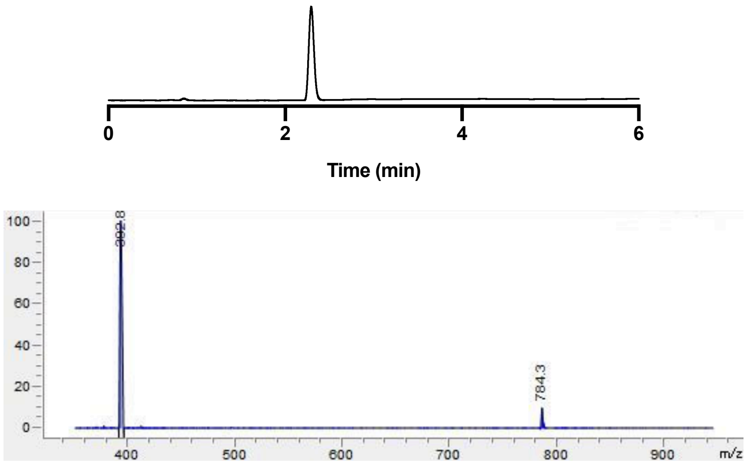

Supplementary Figure 2. HPLC trace and MS (ESI) of purified CDap(PhC8)ANK (**2a**).

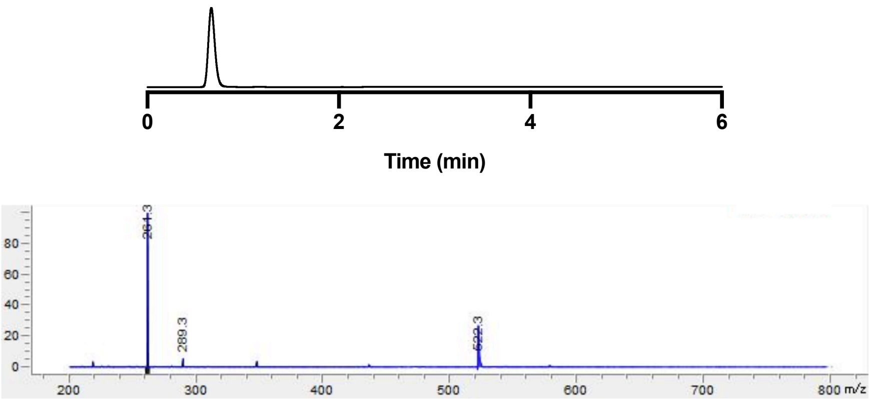

Supplementary Figure 3. HPLC trace and MS (ESI) of purified CDapANK (**2c**).

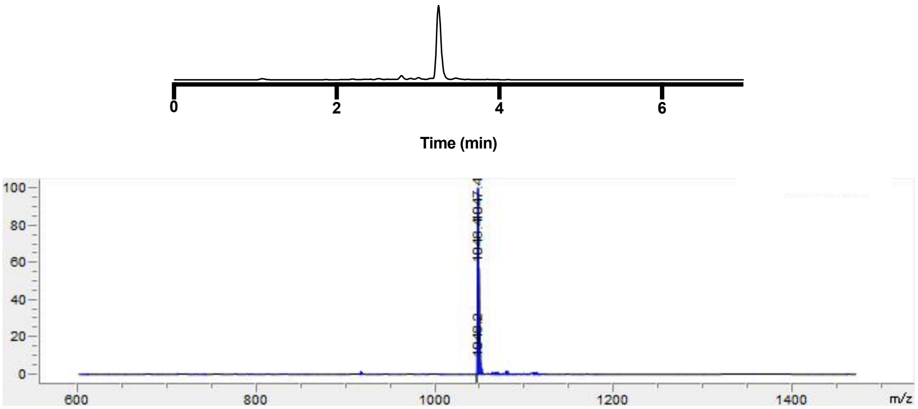

Supplementary Figure 4. HPLC trace and MS (ESI) of purified Boc-Leu-Tyr(^{t}Bu)-Arg(Pbf)-Met-Gly-OH.

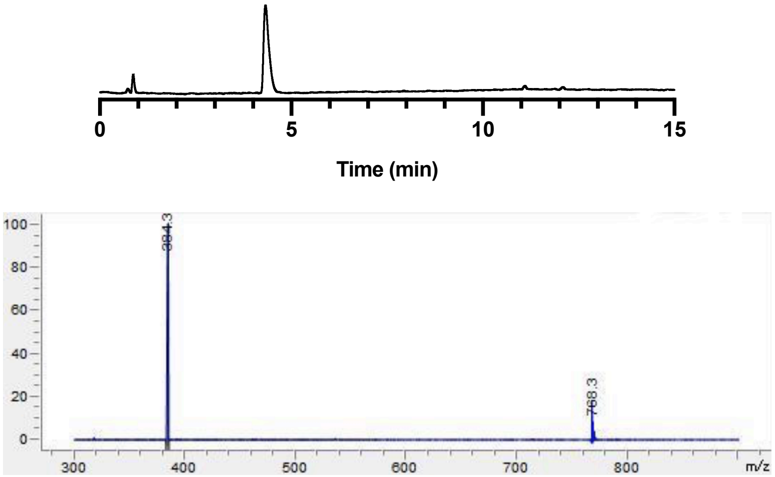

Supplementary Figure 5. HPLC trace and MS (ESI) of purified LYRMG-αCOSC8 (**1a**).

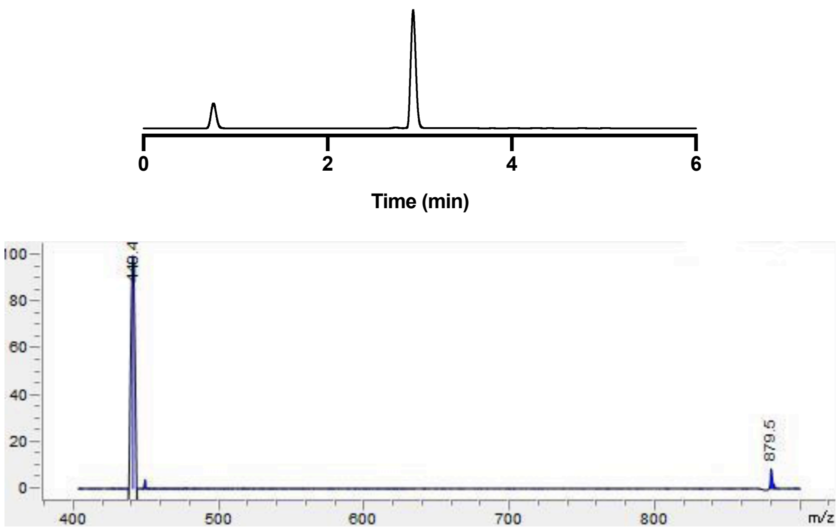

Supplementary Figure 6. HPLC trace and MS (ESI) of purified LYRMG-αCOSC16 (**1b**).

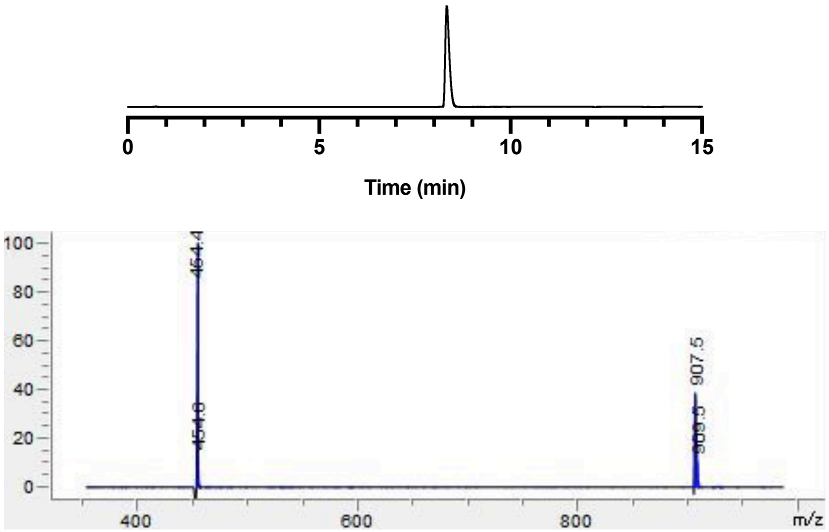

Supplementary Figure 7. HPLC trace and MS (ESI) of purified LYRMG-αCOSC18 (**1c**).

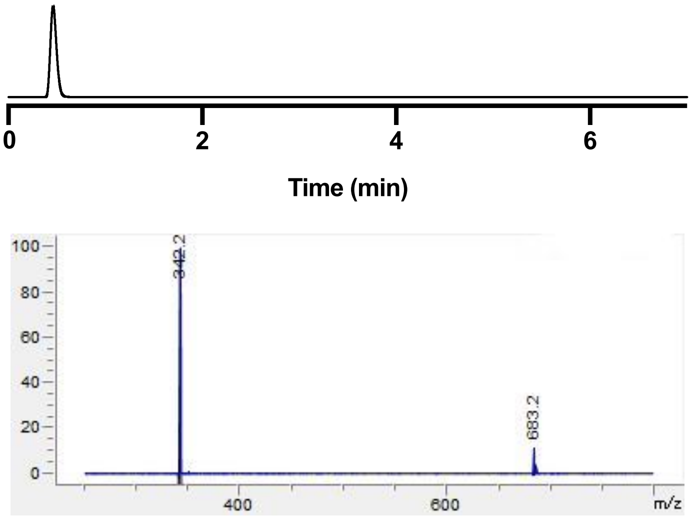

Supplementary Figure 8. HPLC trace and MS (ESI) of purified LYRMG-αCOSC2 (**1d**).

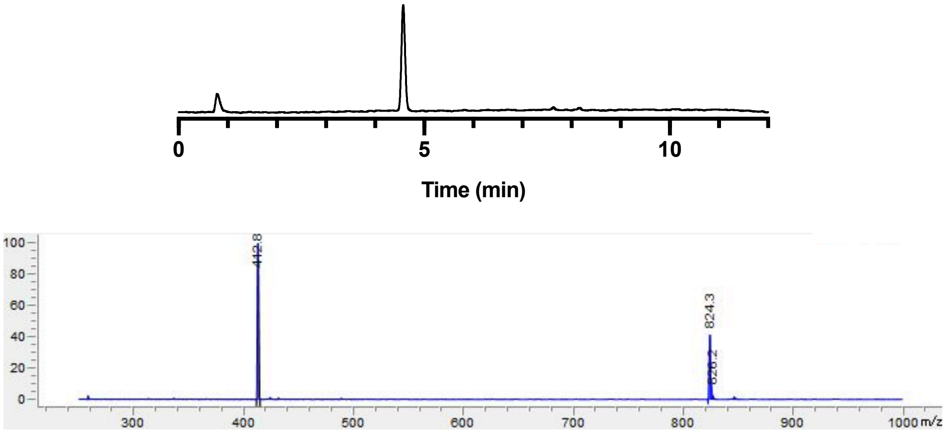

Supplementary Figure 9. HPLC trace and MS (ESI) of purified LYRMN-αCOSC8 (**5a**).

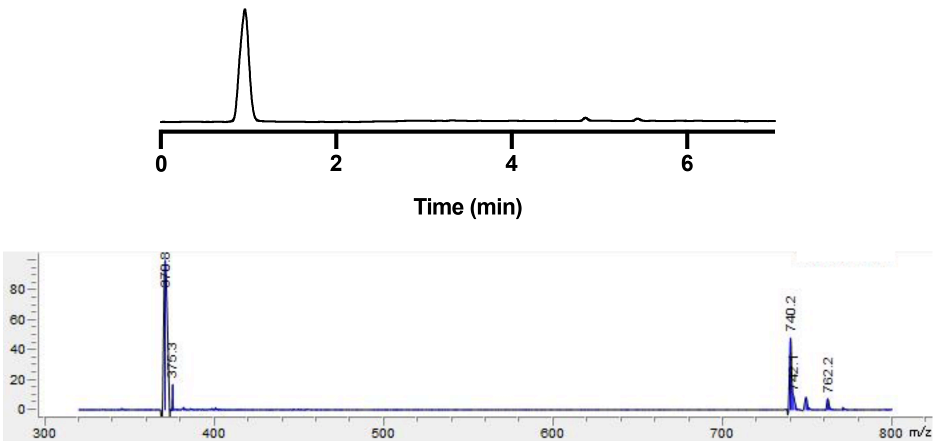

Supplementary Figure 10. HPLC trace and MS (ESI) of purified LYRMN-αCOSC2 (**5b**).

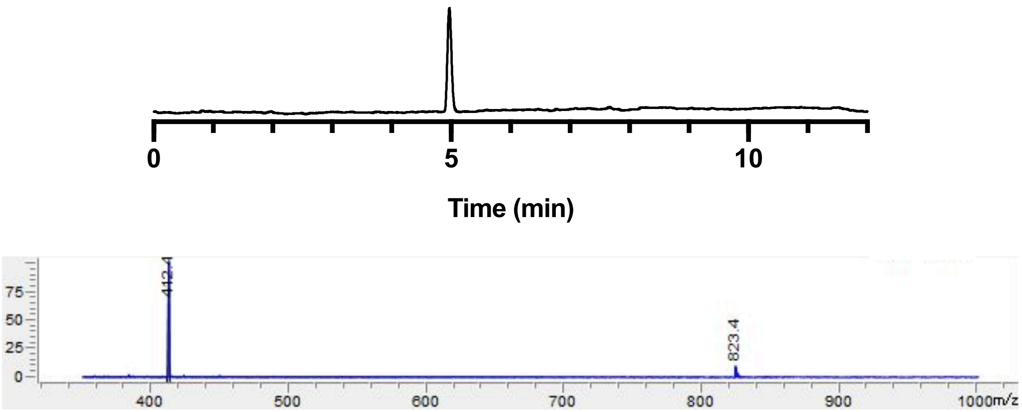

Supplementary Figure 11. HPLC trace and MS (ESI) of purified LYRML-αCOSC8 (**6a**).

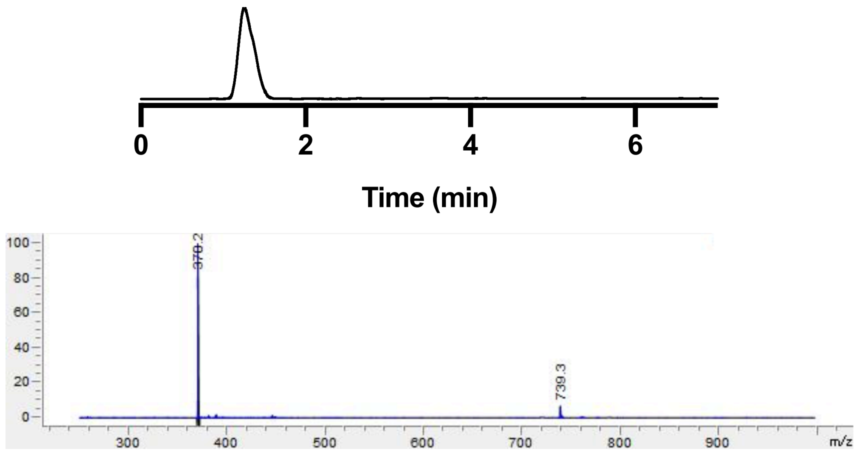

Supplementary Figure 12. HPLC trace and MS (ESI) of purified LYRML-αCOSC2 (**6b**).

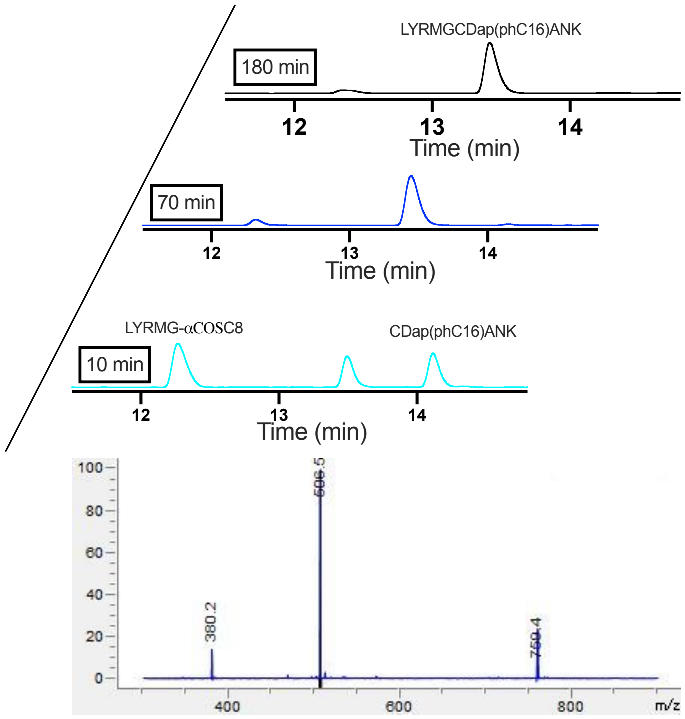

Supplementary Figure 13. HPLC traces of NCL and MS (ESI) of LYRMGCDap(PhC16)ANK (**3b**).

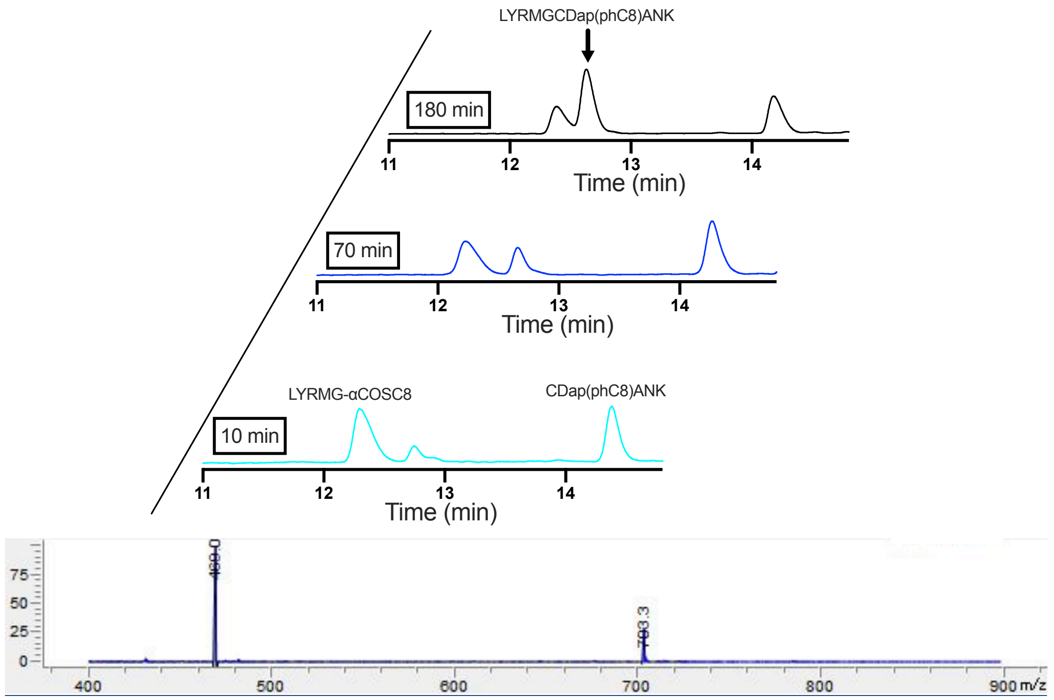

Supplementary Figure 14. HPLC traces of NCL and MS (ESI) of LYRMGCDap(PhC8)ANK (**3a**).

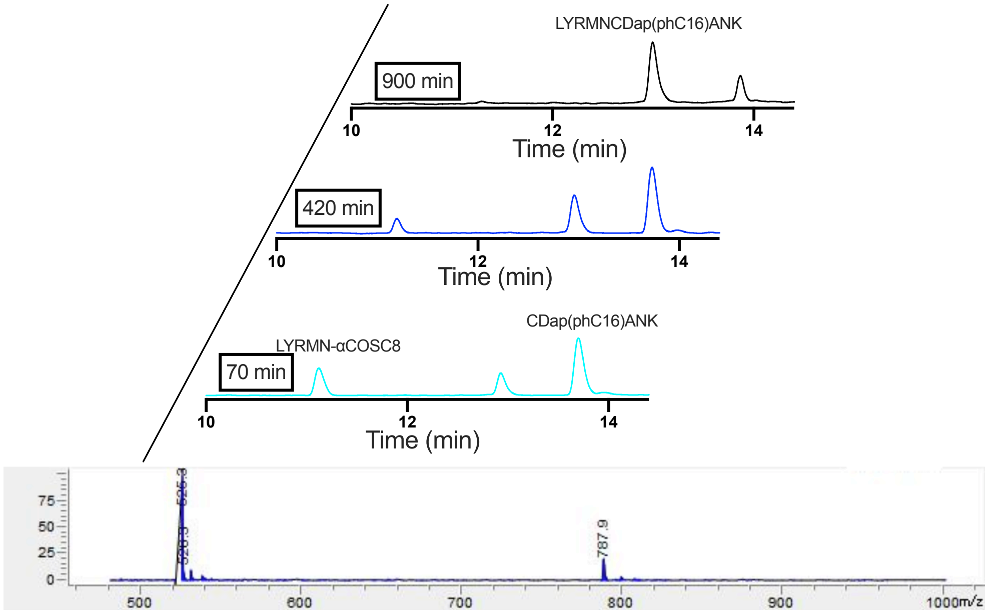

Supplementary Figure 15. HPLC traces and MS (ESI) of LYRMNCDap(PhC16)ANK (**7**).

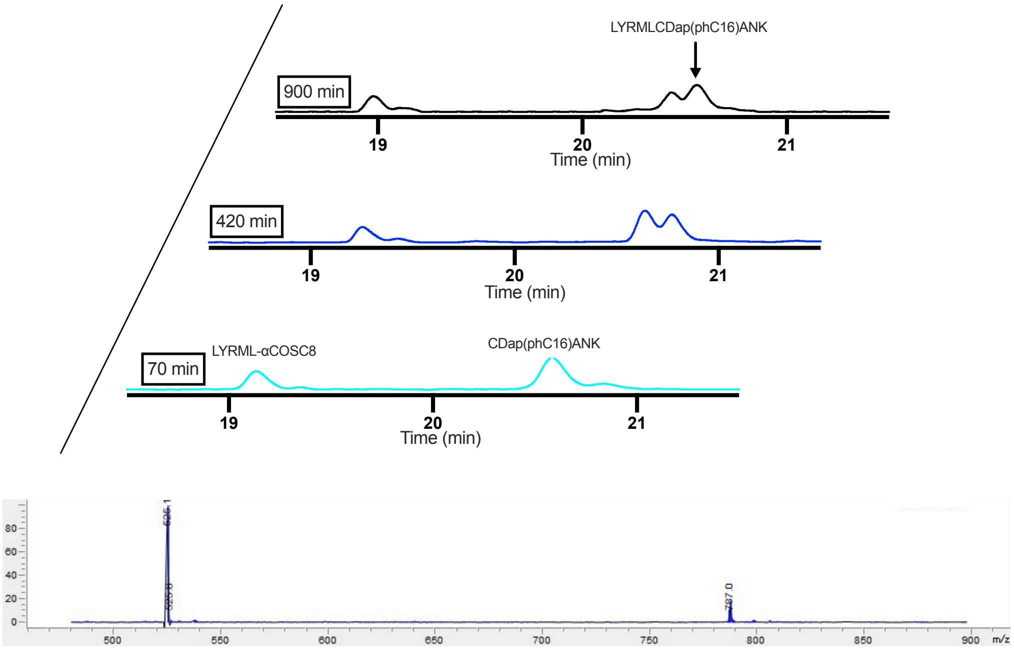

Supplementary Figure 16. HPLC traces and MS (ESI) of LYRMLCDap(PhC16)ANK (**8**).

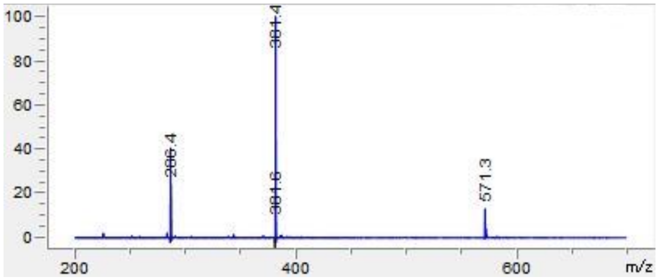

Supplementary Figure 17. MS (ESI) of LYRMGCDapANK (**4**).

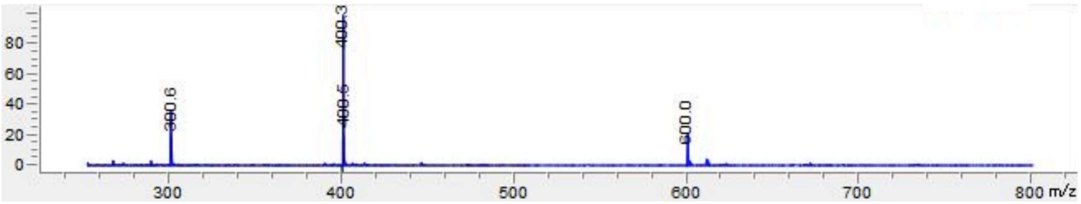

Supplementary Figure 18. MS (ESI) of LYRMNCDapANK (**9**).

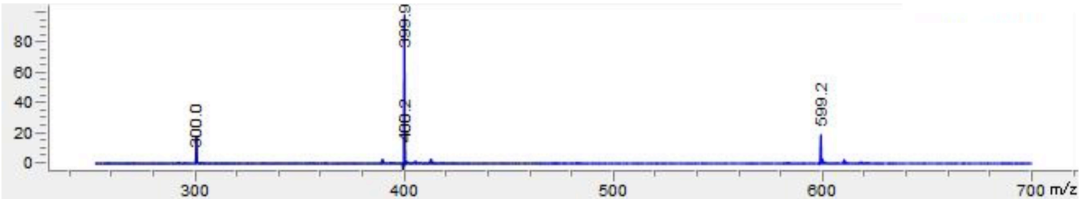

Supplementary Figure 19. MS (ESI) of LYRMLCDapANK (**10**).

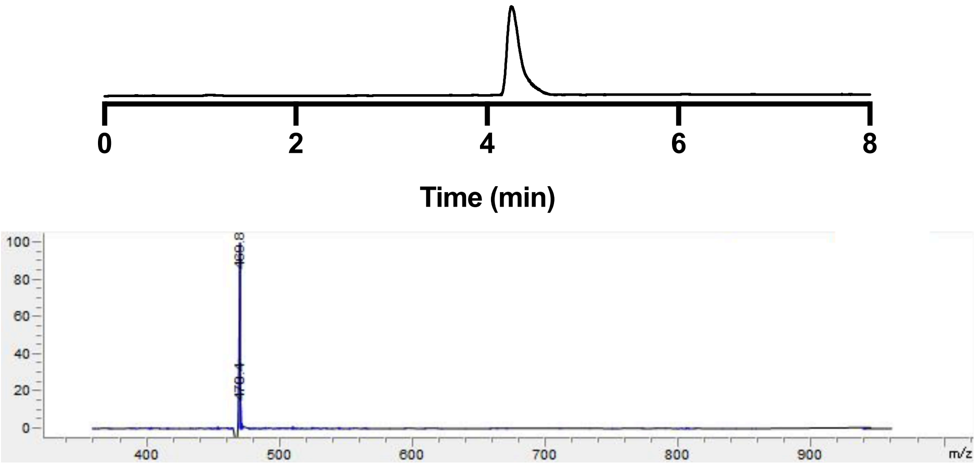

Supplementary Figure 20. HPLC trace and MS (ESI) of purified CK(PhC16)ANK (**S4**).

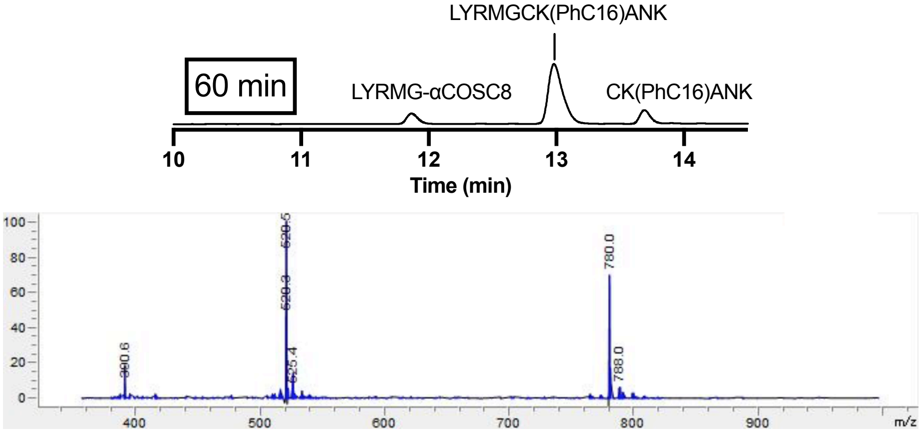

Supplementary Figure 21. HPLC trace of NCL reaction (after 60 min) and MS (ESI) of LYRMGCK(PhC16)ANK (**S5**).

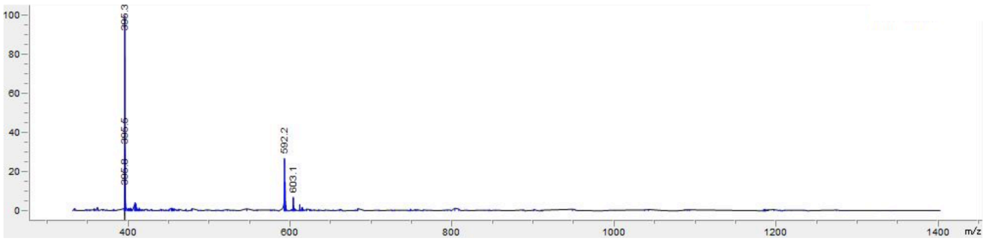

Supplementary Figure 22. MS (ESI) of LYRMNCKANK (**S6**).

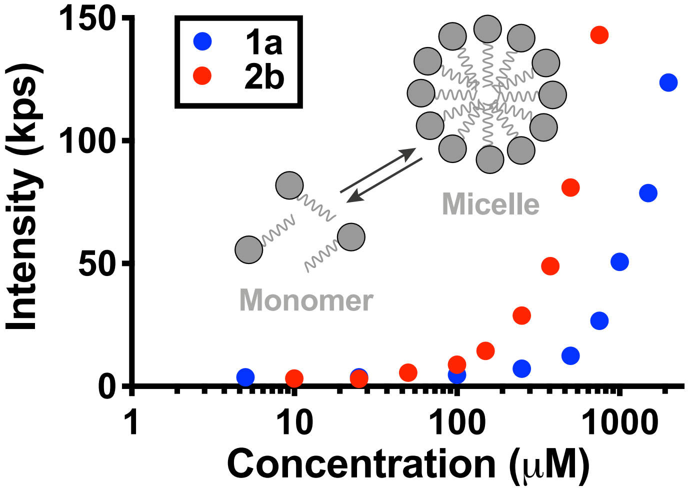

Supplementary Figure 23 Determination of critical micelle concentrations (CMCs) of LYRMG-αCOSC8 (**1a**) and the CDap(PhC16)ANK (**2b**) using dynamic light scattering (DLS). The CMCs of **1a** and **2b** were estimated to be 380 μM and 130 μM, respectively.

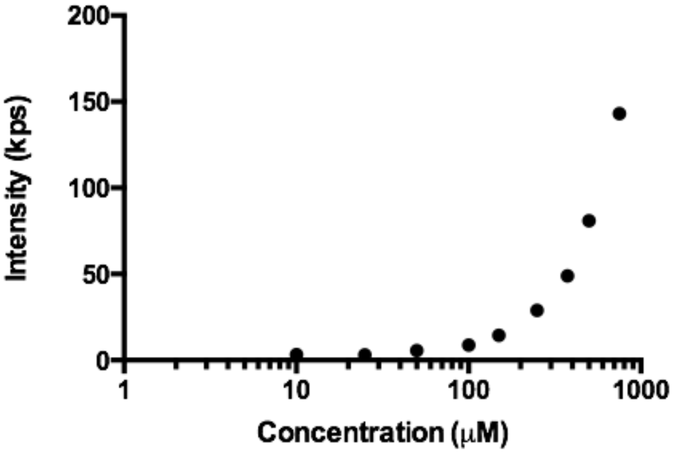

Supplementary Figure 24. Determination of critical micelle concentration (CMC) of CDap(PhC8)ANK (**2a**).

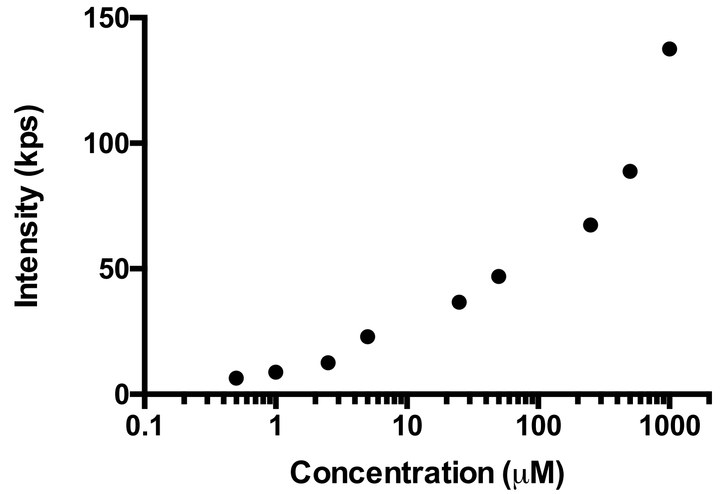

Supplementary Figure 25. Determination of critical micelle concentration (CMC) of LYRMG-αCOSC16 (**1b**).

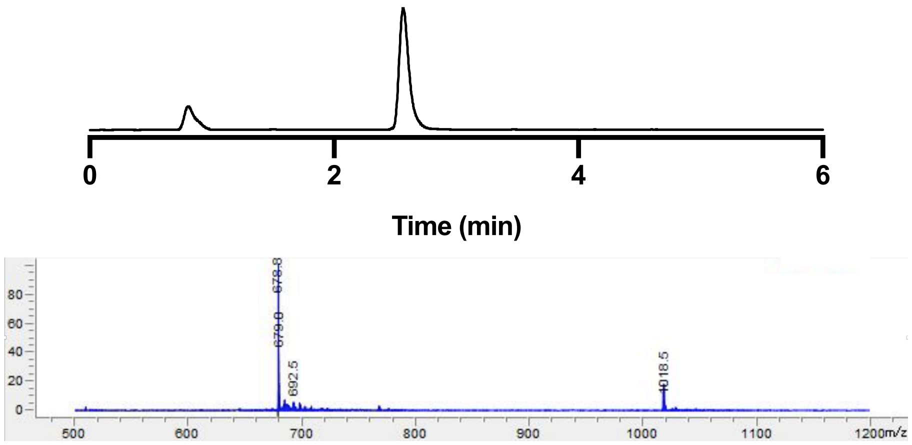

Supplementary Figure 26. HPLC trace and MS (ESI) of purified CK(PhC16)KFGKAFVGEIMNS (**12**).

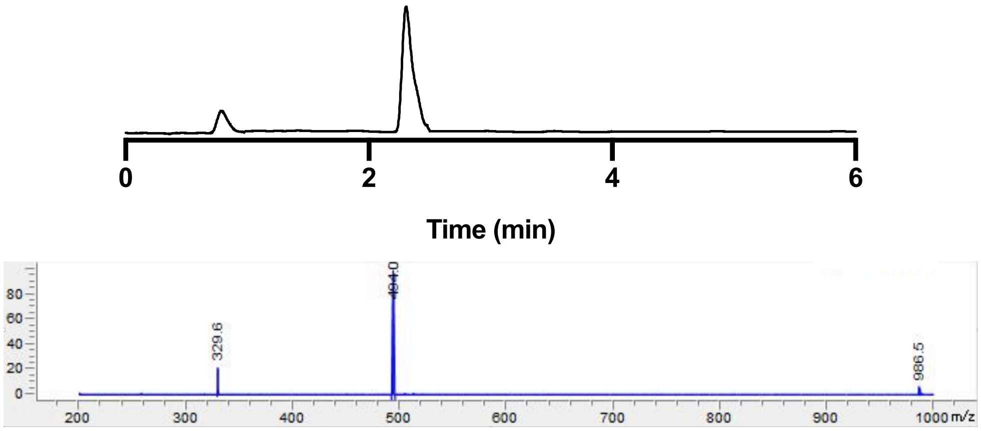

Supplementary Figure 27. HPLC trace and MS (ESI) of purified GIGKFLHS-αCOSC8 (**11**).

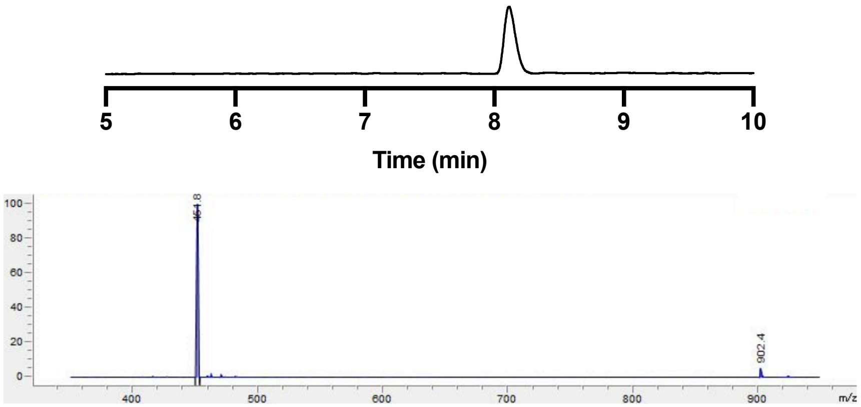

Supplementary Figure 28. HPLC trace and MS (ESI) of purified GIGKFLHS-αCOSC2 (**S8**).

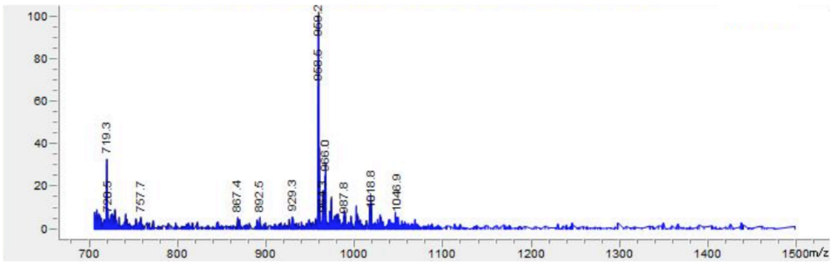

Supplementary Figure 29. MS (ESI) of GIGKFLHSCK(PhC16)KFGKAFVGEIMNS (**13**).

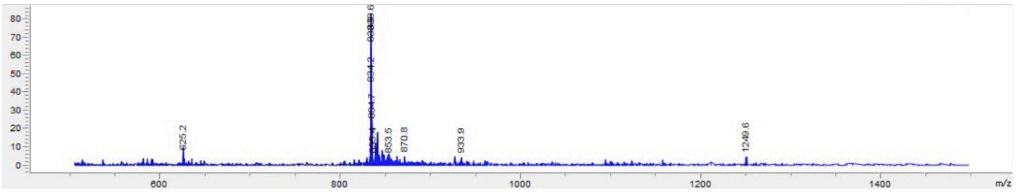

Supplementary Figure 30. MS (ESI) of GIGKFLHSCKKFGKAFVGEIMNS (**14**).

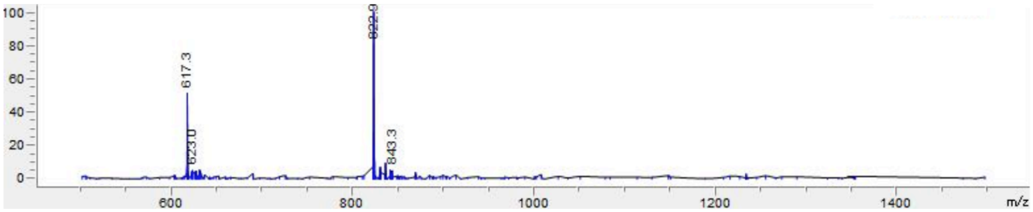

Supplementary Figure 31. MS (ESI) of GIGKFLHSAKKFGKAFVGEIMNS (Magainin 2, **15**).

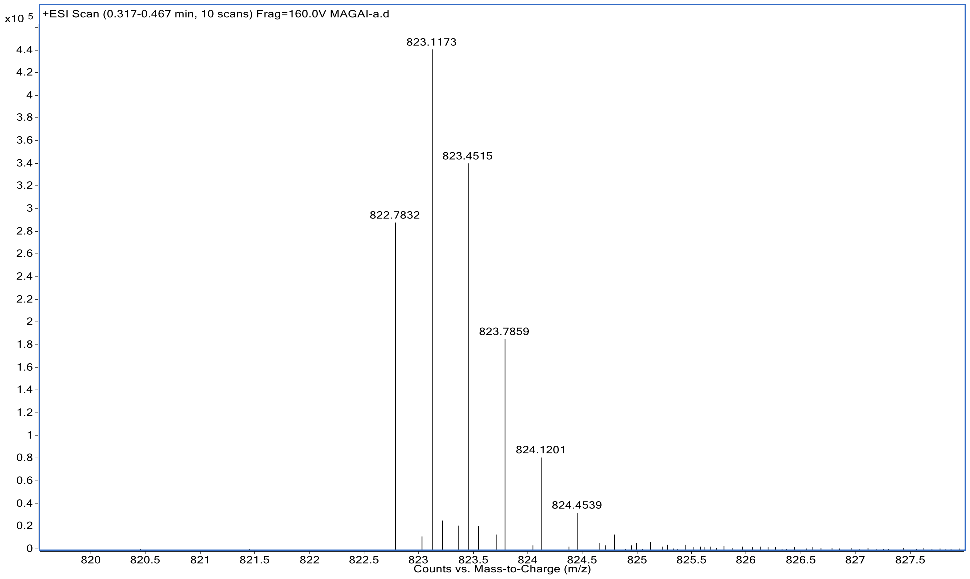

Mass Measured	Theo. Mass	Delta (ppm)	Composition
822.7832	822.7824	1.0	$[C_{114}H_{183}N_{30}O_{29}S]^{3+}$

Supplementary Figure 32. HRMS (ESI-TOF) of GIGKFLHSAKKFGKAFVGEIMNS (Magainin 2, **15**).

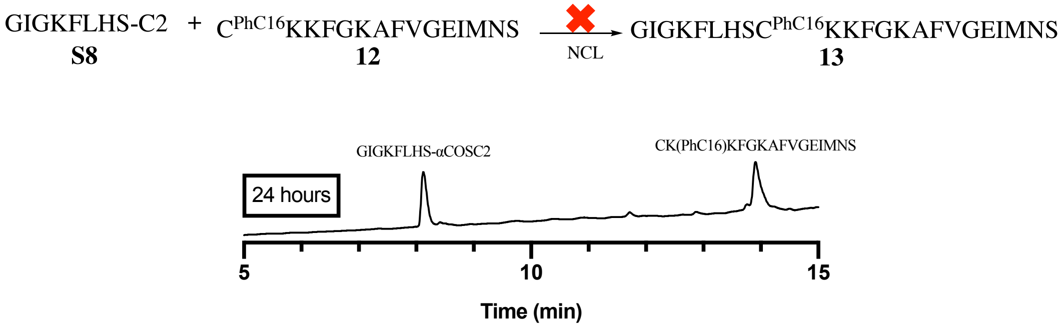

Supplementary Figure 33. HPLC trace of NCL between **S8** and **12** after 24 h (Control reaction).

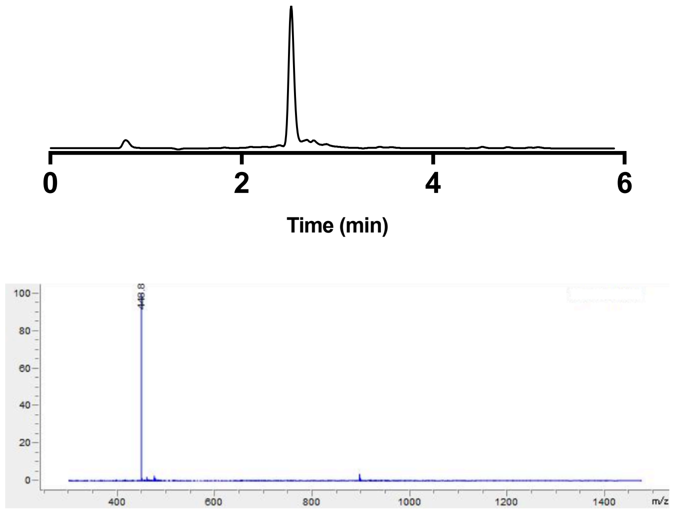

Supplementary Figure 34. HPLC trace and MS (ESI) of purified CADap(PhC16)NK (**S10**).

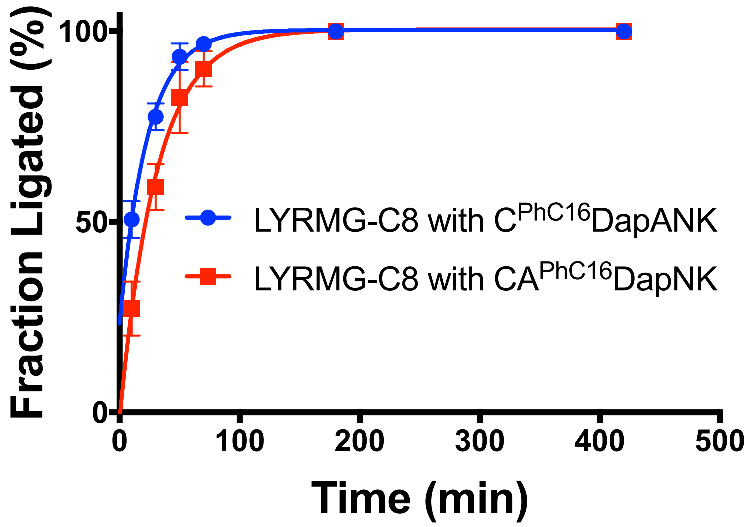

Supplementary Figure 35. Kinetic measurment of NCL of LYRMG-αCOSC8 (**1a**) (1 mM) with the CDap(PhC16)ANK (**2b**) and CADap(PhC16)NK (**S10**).

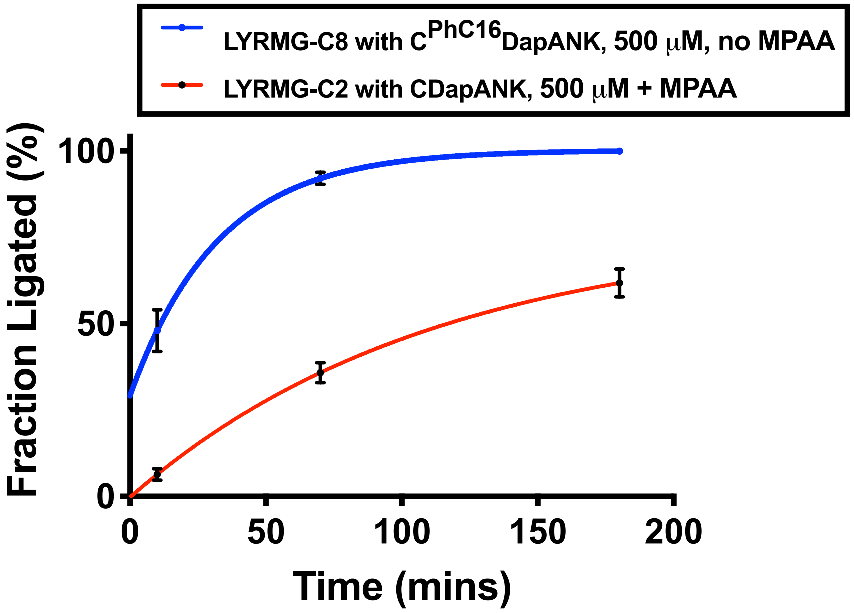

Supplementary Figure 36. Kinetic measurment of NCL of LYRMG-αCOSC8 (**1a**, 500 μM) with CDap(PhC16)ANK (**2b**, 500 μM) and NCL of LYRMG-αCOSC2 (**1d**, 500 μM) and CDapANK (**2c**, 500 μM) with MPAA additive.

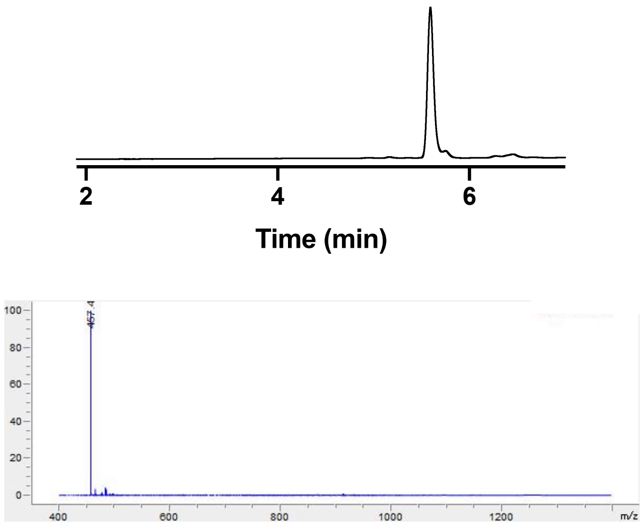

Supplementary Figure 37. HPLC trace and MS (ESI) of purified CK(PhC16)ANC (**S12**).

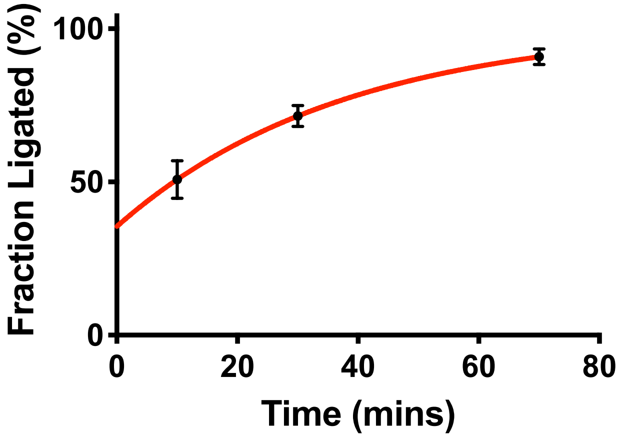

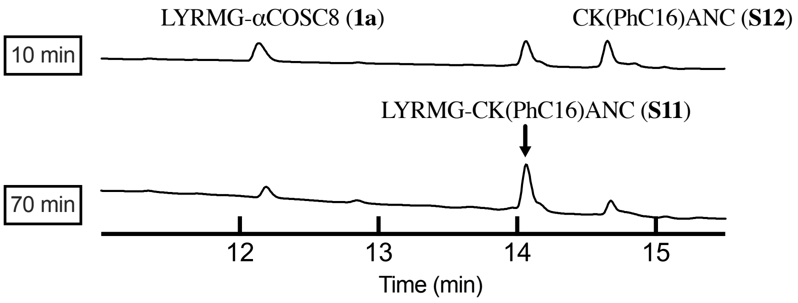

The HPLC elution of peak (**S11**) with retention time t_R = 14.05 min was collected and subjected to MS-MS analysis (Supplementary Fig. 39)

Supplementary Figure 38. HPLC traces and kinetic measurement of NCL between LYRMG-αCOSC8 (**1a**, 1 mM) and CK(PhC16)ANC (**S12**, 1mM).

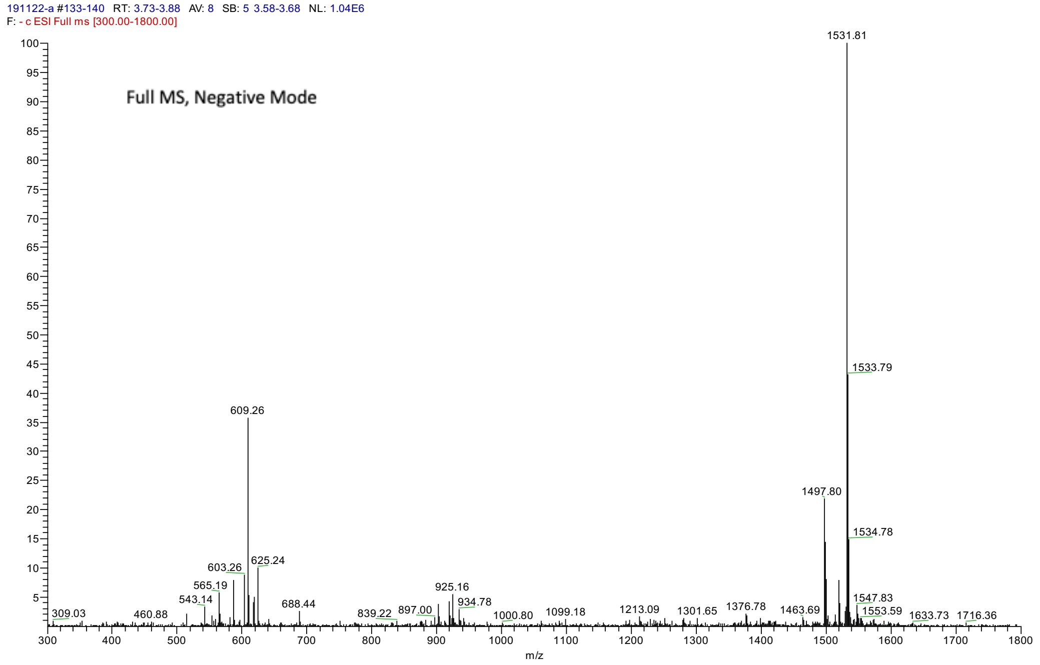

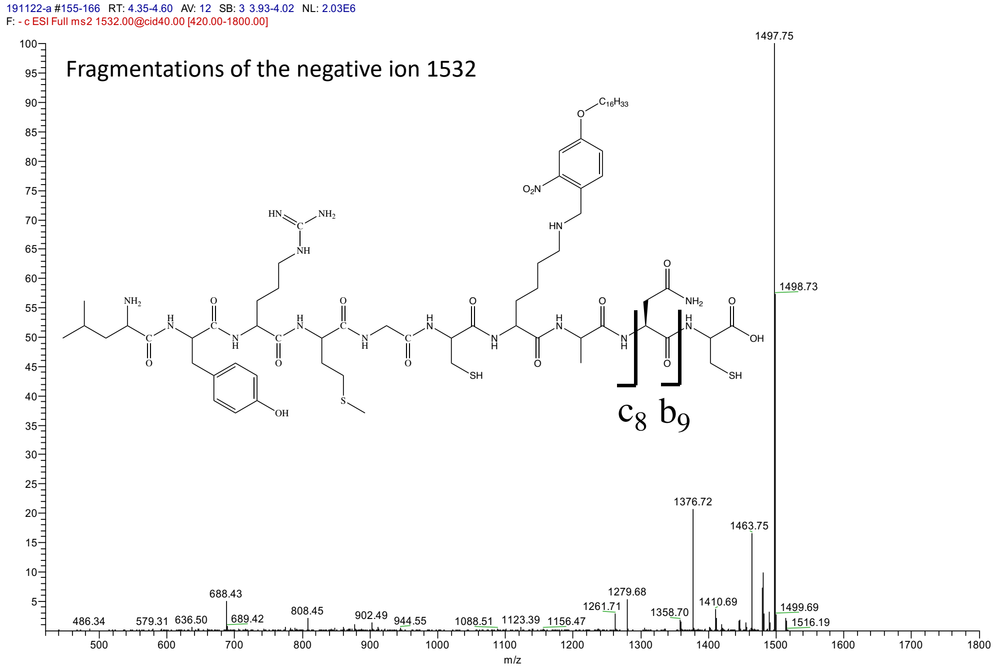

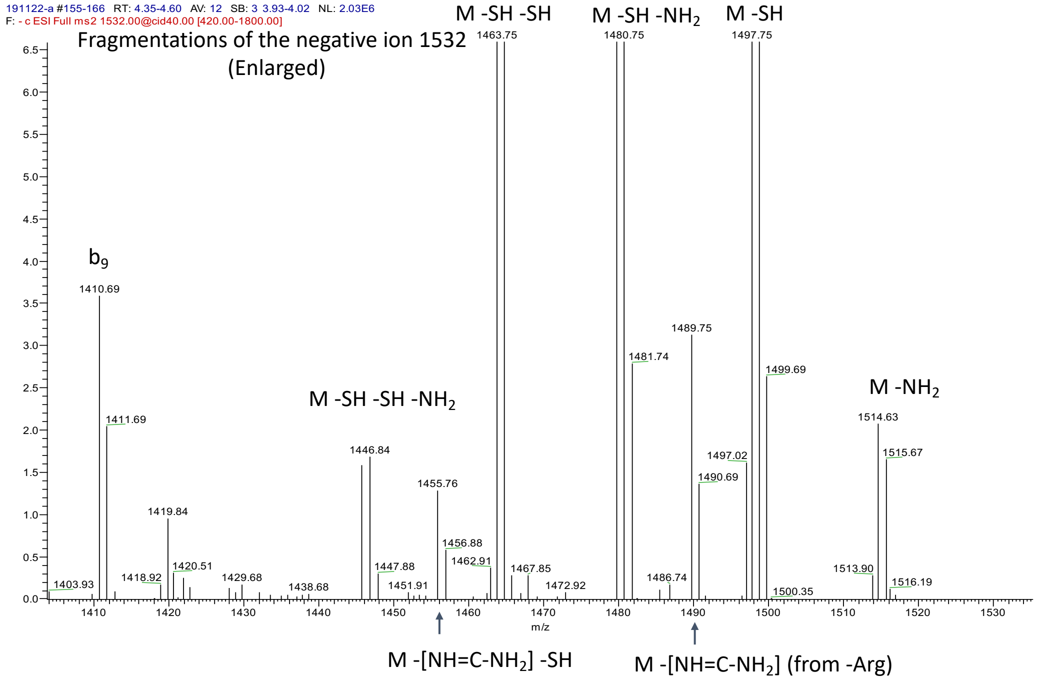

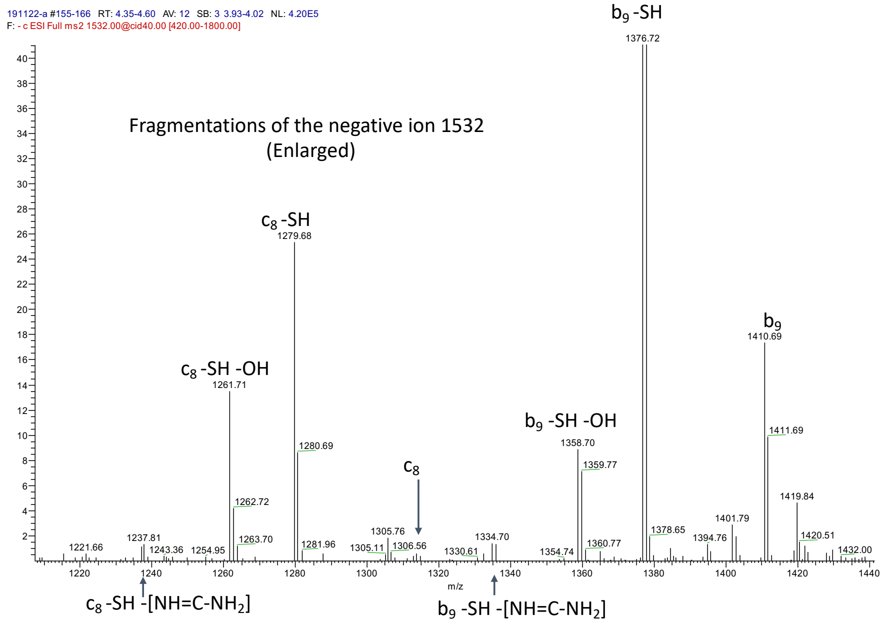

Supplementary Figure 39. MS-MS analysis spectra of ligation product LYRMGCK(PhC16)ANC (**S11**).

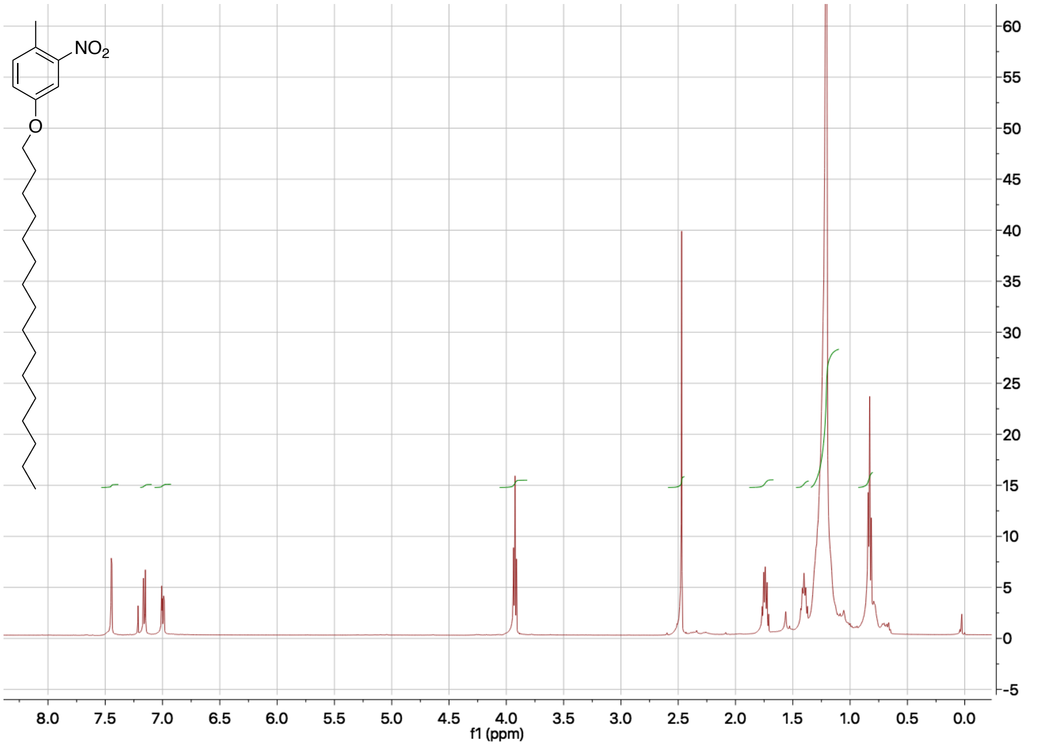

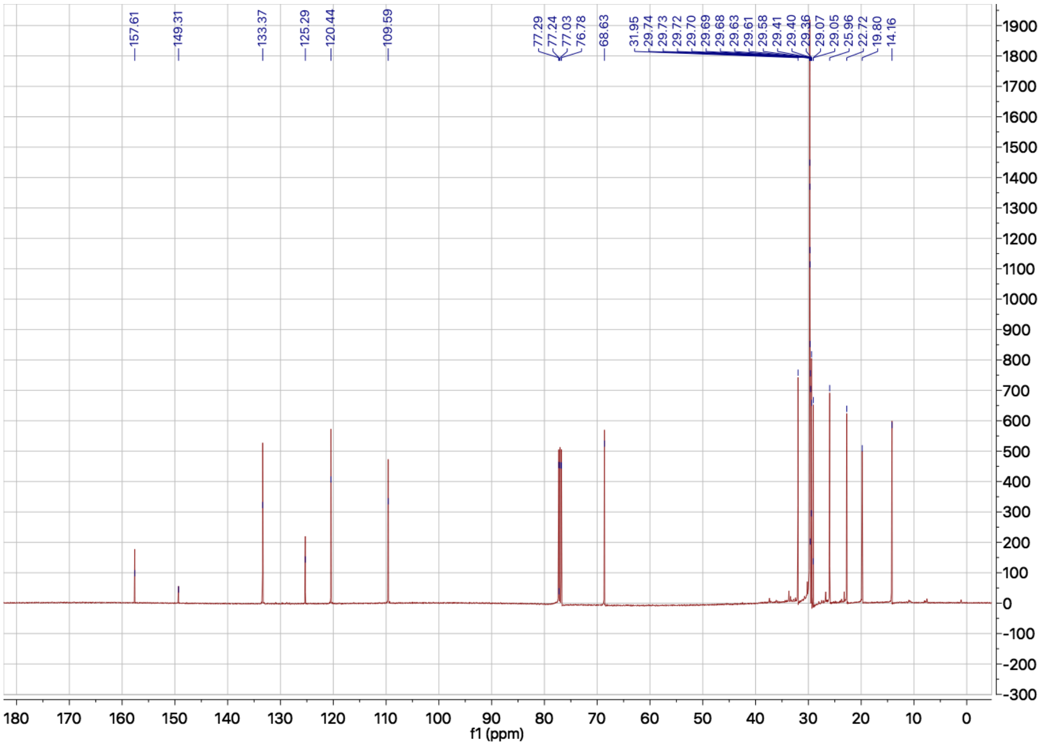

Supplementary Figure 40. ^{1}H NMR ($CDCl_3$, 500.13 MHz) (*top*) and ^{13}C NMR ($CDCl_3$, 125.77 MHz) (*bottom*) spectra of 4-(hexadecyloxy)-1-methyl-2-nitrobenzene (**S1a**).

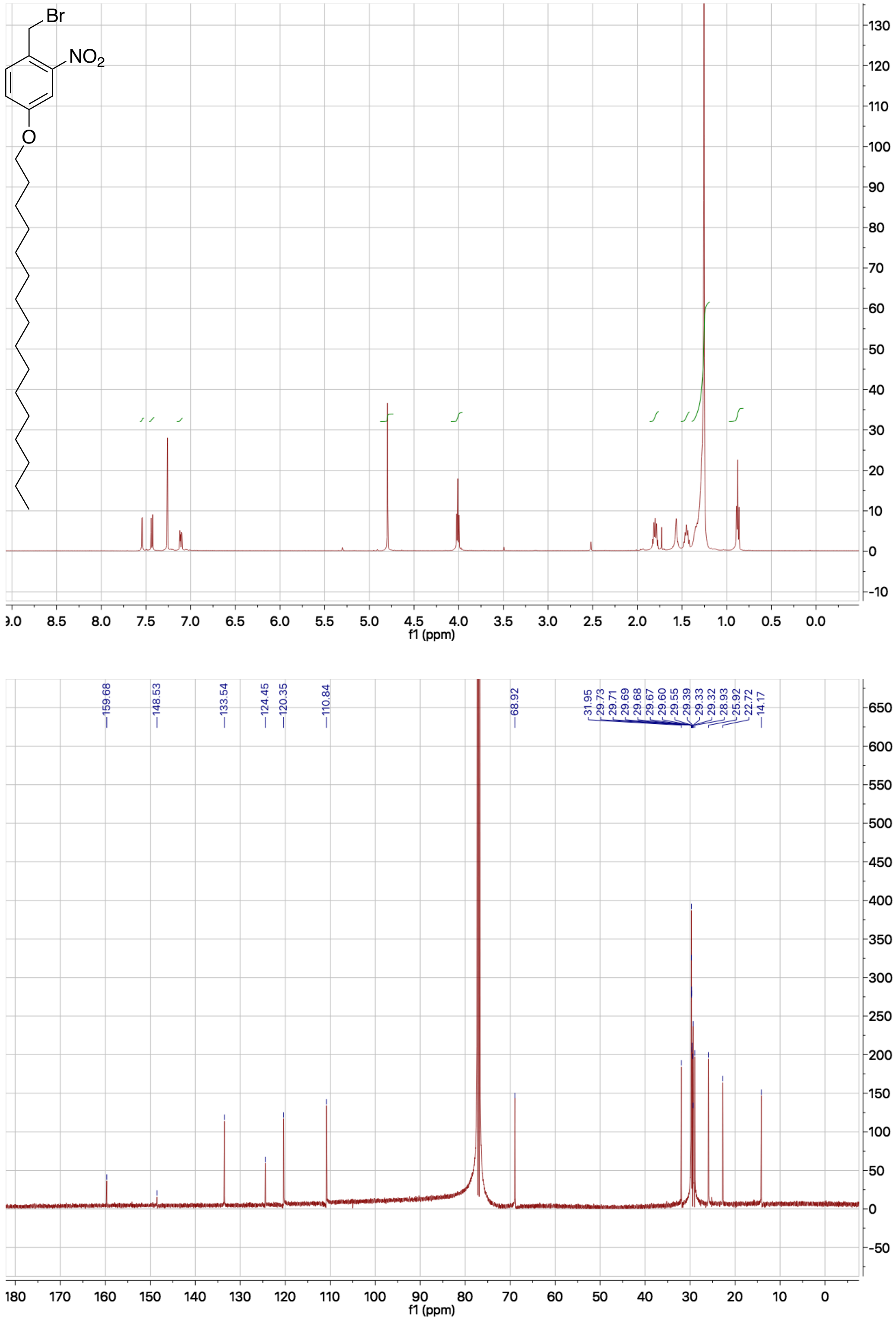

Supplementary Figure 41. ^{1}H NMR ($CDCl_3$, 500.13 MHz) (*top*) and ^{13}C NMR ($CDCl_3$, 125.77 MHz) (*bottom*) spectra of 1-(bromomethyl)-4-(hexadecyloxy)-2-nitrobenzene (**S2a**).

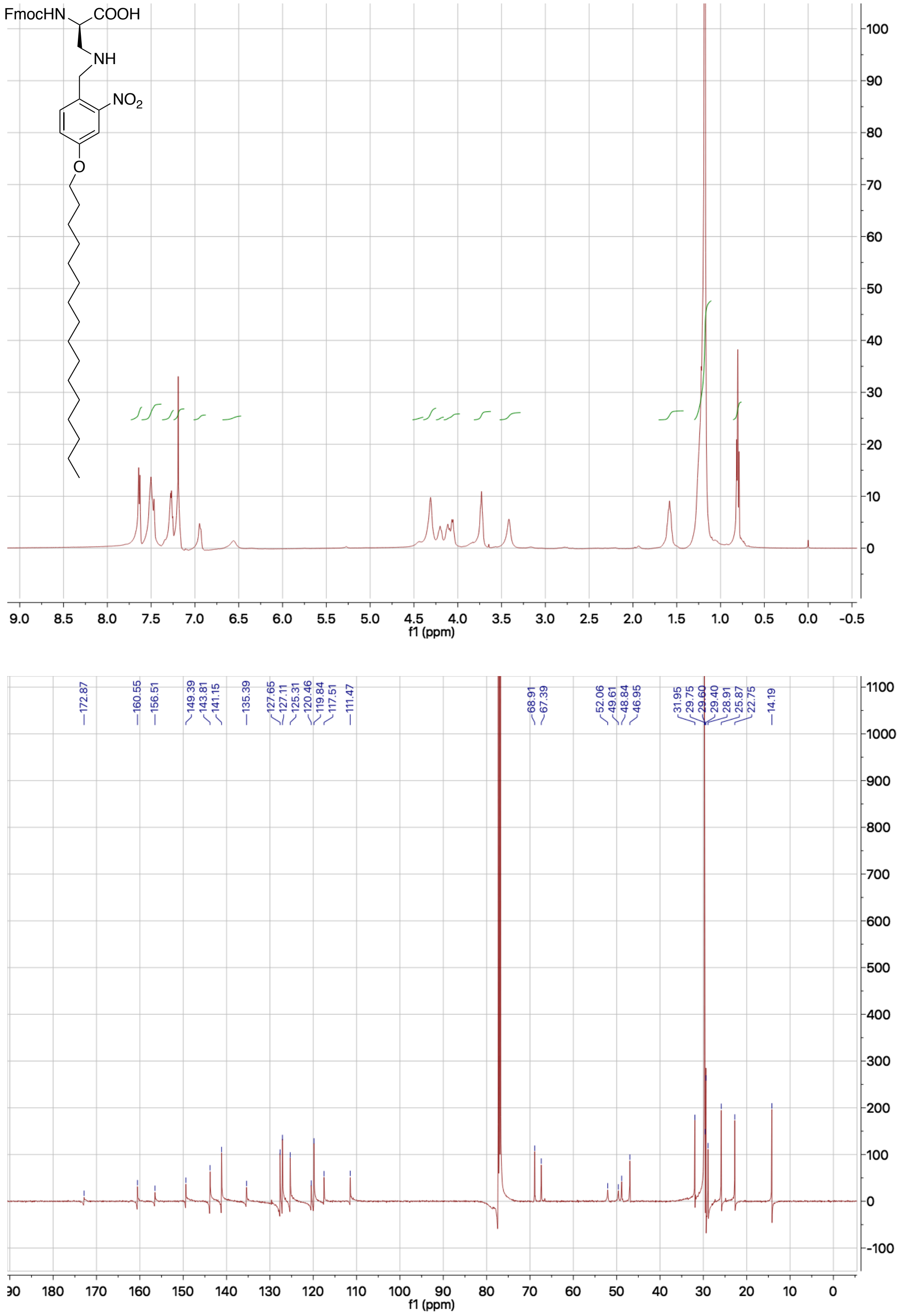

Supplementary Figure 42. ^{1}H NMR ($CDCl_3$, 500.13 MHz) (*top*) and ^{13}C NMR ($CDCl_3$, 125.77 MHz) (*bottom*) spectra of (R)-2-((((9H-fluoren-9-yl)methoxy)carbonyl)amino)-3-((4-(hexadecyloxy)-2-nitro-benzyl)amino)propanoic acid (**S3a**).

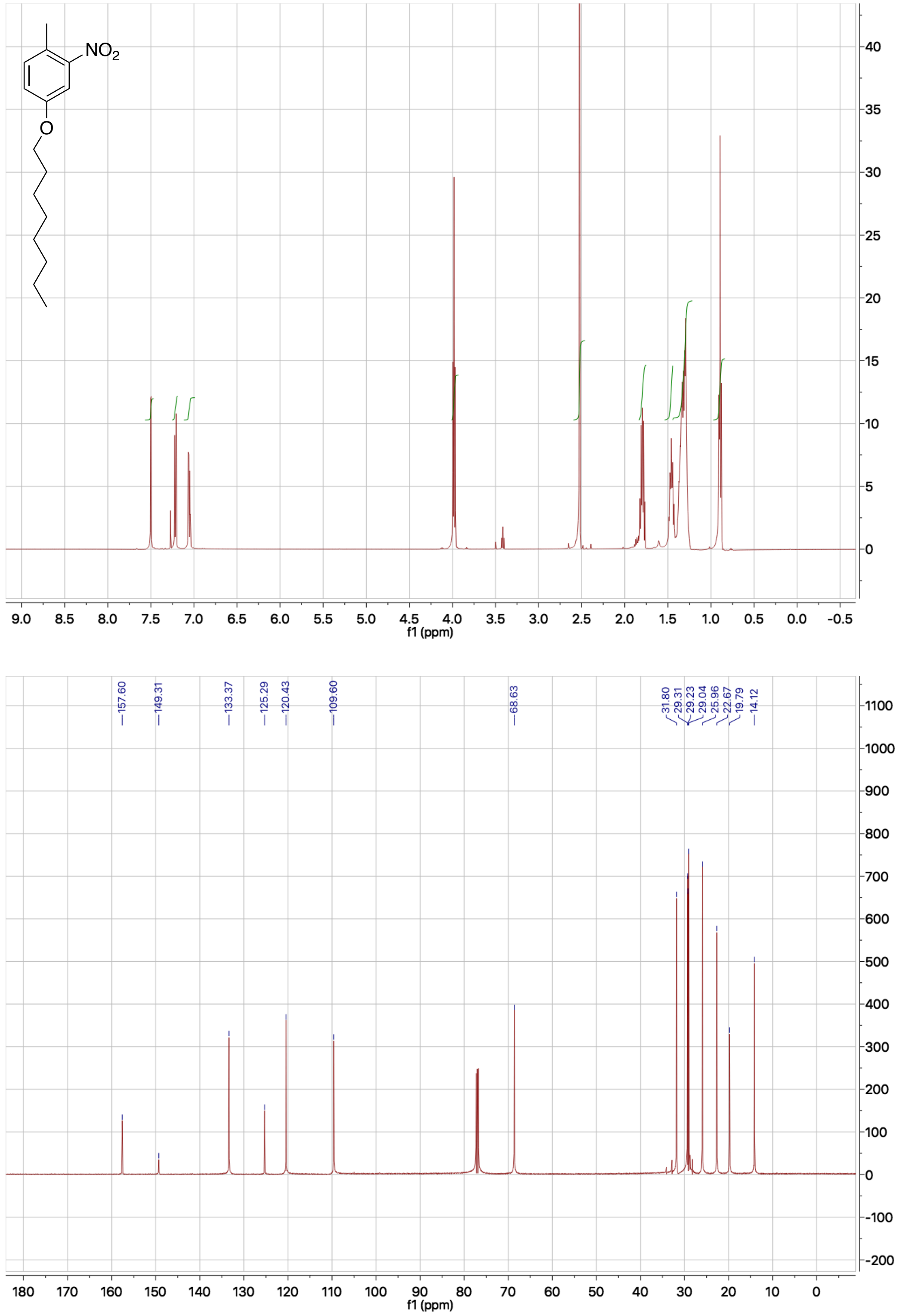

Supplementary Figure 43. ^{1}H NMR ($CDCl_3$, 500.13 MHz) (*top*) and ^{13}C NMR ($CDCl_3$, 125.77 MHz) (*bottom*) spectra of 1-methyl-2-nitro-4-(octyloxy)benzene (**S1b**).

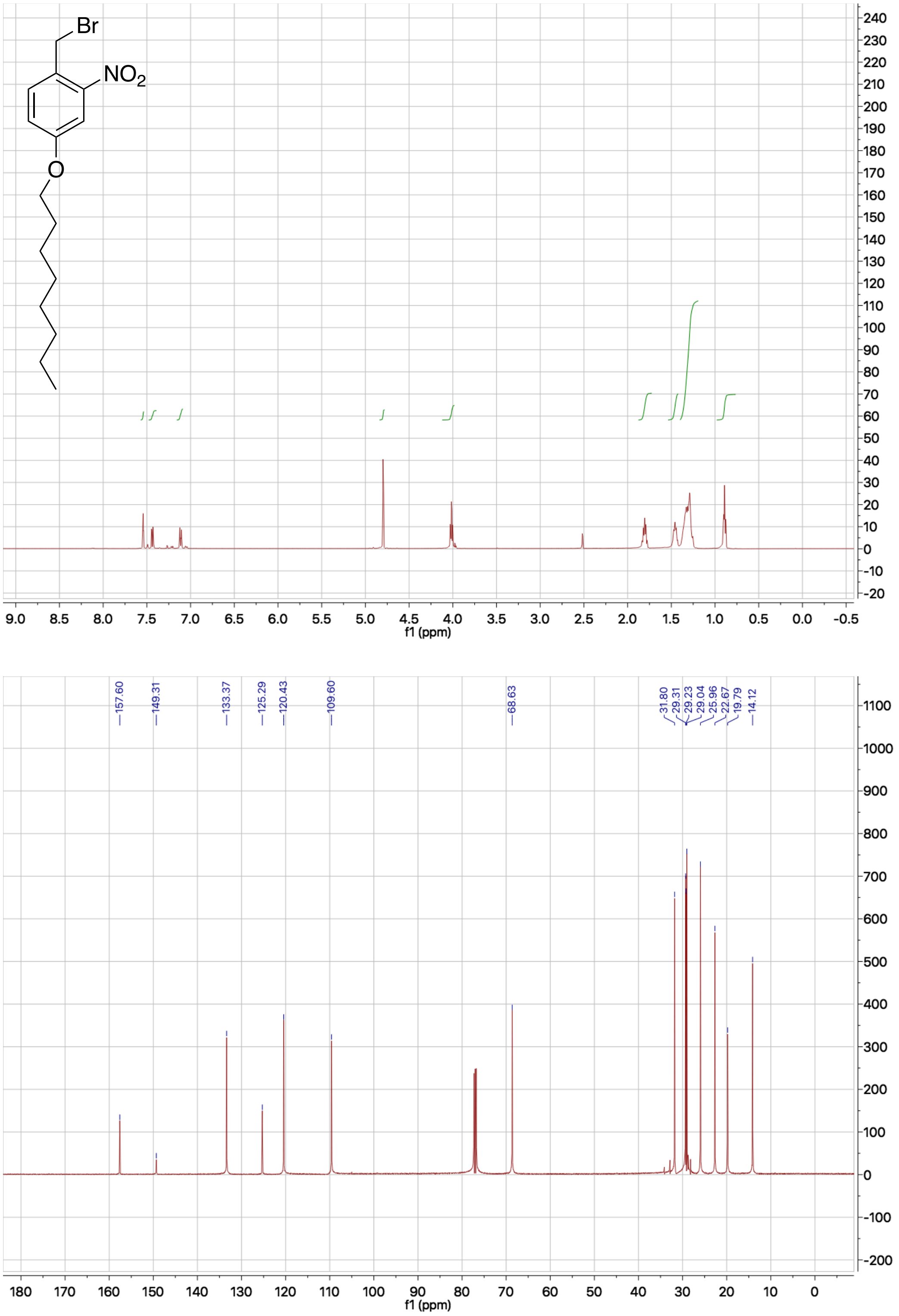

Supplementary Figure 44. ^{1}H NMR ($CDCl_3$, 500.13 MHz) (*top*) and ^{13}C NMR ($CDCl_3$, 125.77 MHz) (*bottom*) spectra of 1-(bromomethyl)-2-nitro-4-(octyloxy)benzene (**S2b**).

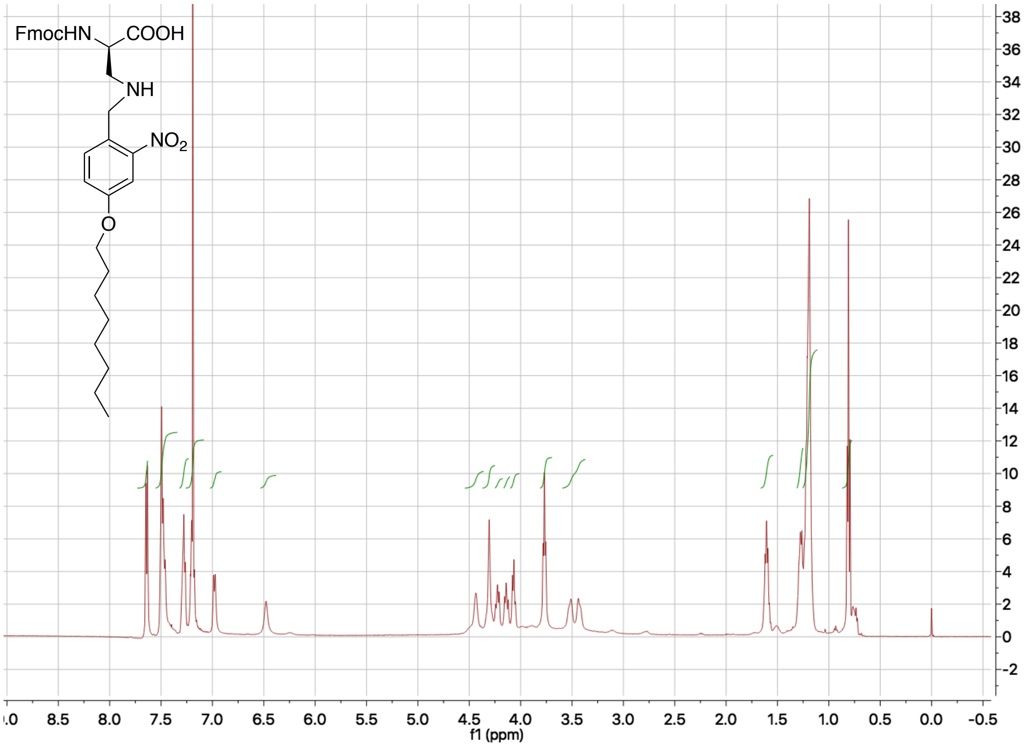

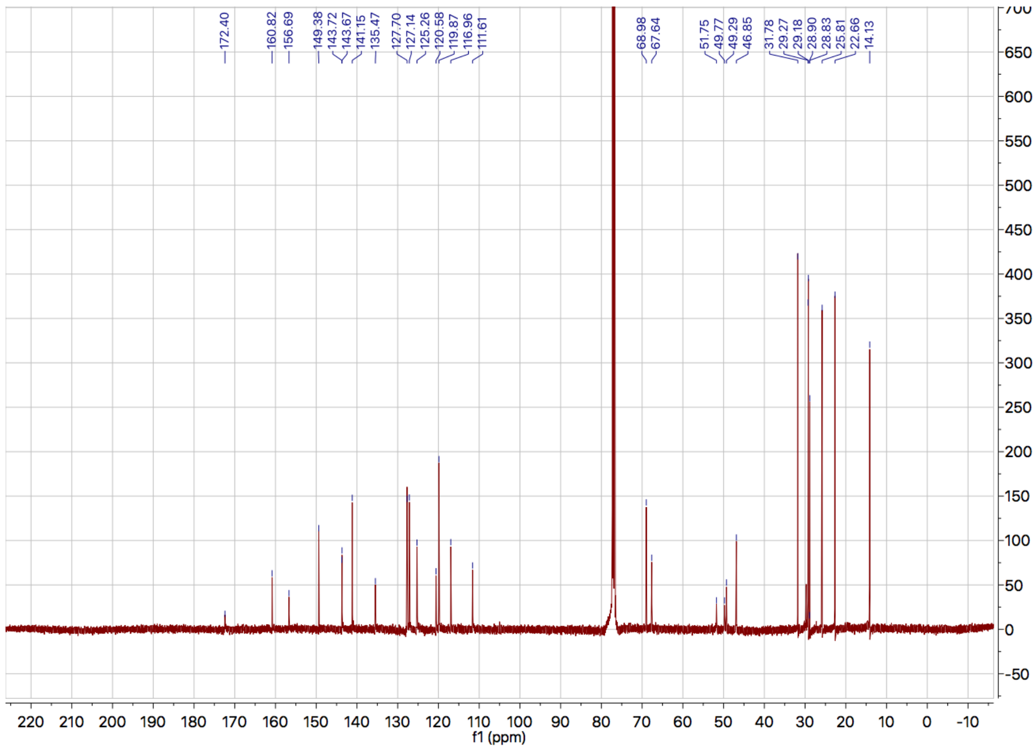

Supplementary Figure 45. ^{1}H NMR ($CDCl_3$, 500.13 MHz) (*top*) and ^{13}C NMR ($CDCl_3$, 125.77 MHz) (*bottom*) spectra of (R)-2-((((9H-fluoren-9-yl)methoxy)carbonyl)amino)-3-((2-nitro-4-(octyloxy)benzyl)-amino)propanoic acid (**S3b**).

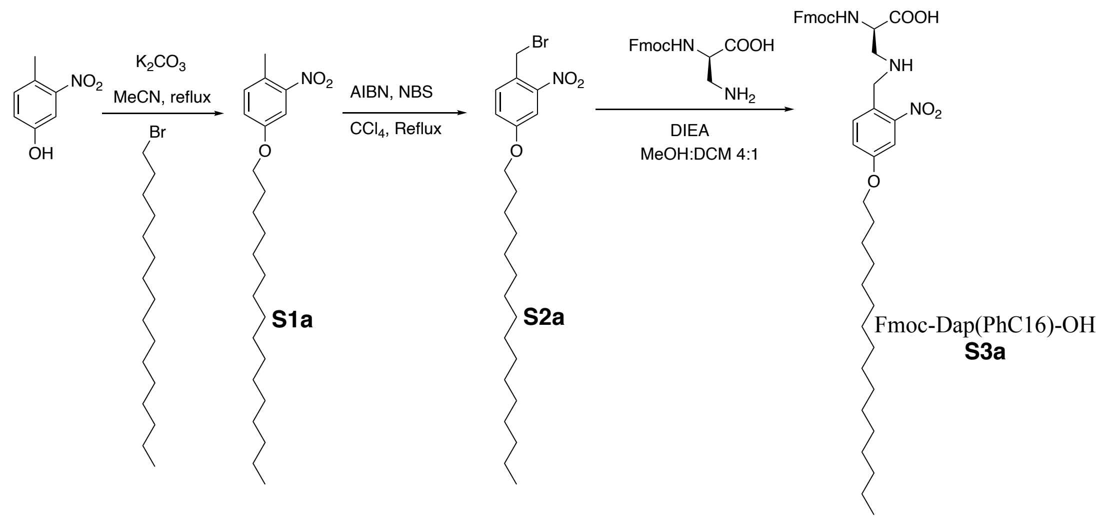

Supplementary Figure 46. Synthesis of Fmoc-Dap(PhC16)-OH (**S3a**).

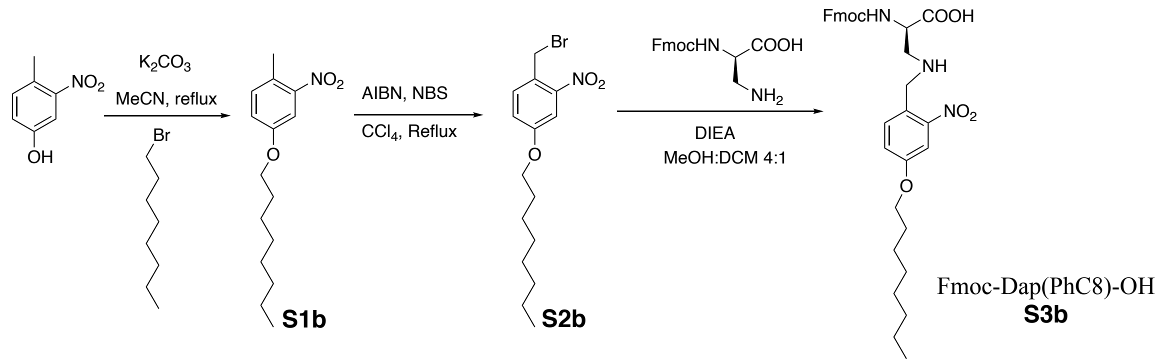

Supplementary Figure 47. Synthesis of Fmoc-Dap(PhC8)-OH (**S3b**).

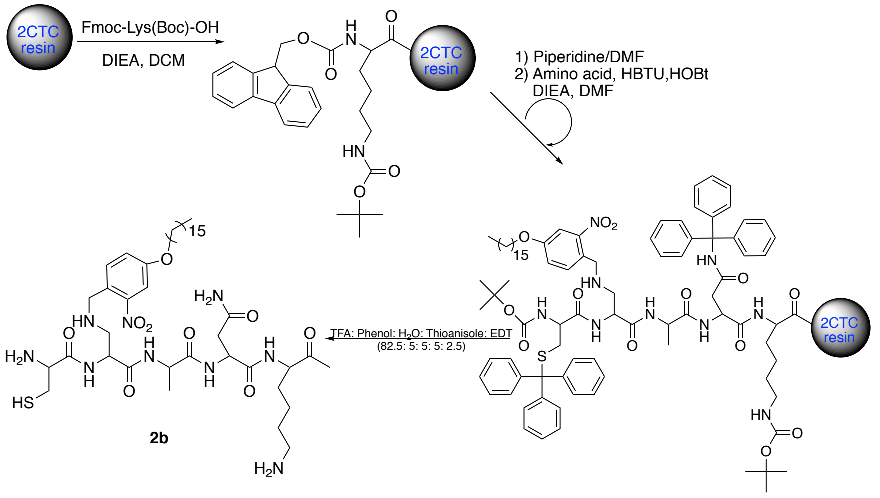

Supplementary Figure 48. Synthesis of the pentapeptide CDap(PhC16)ANK (**2b**).

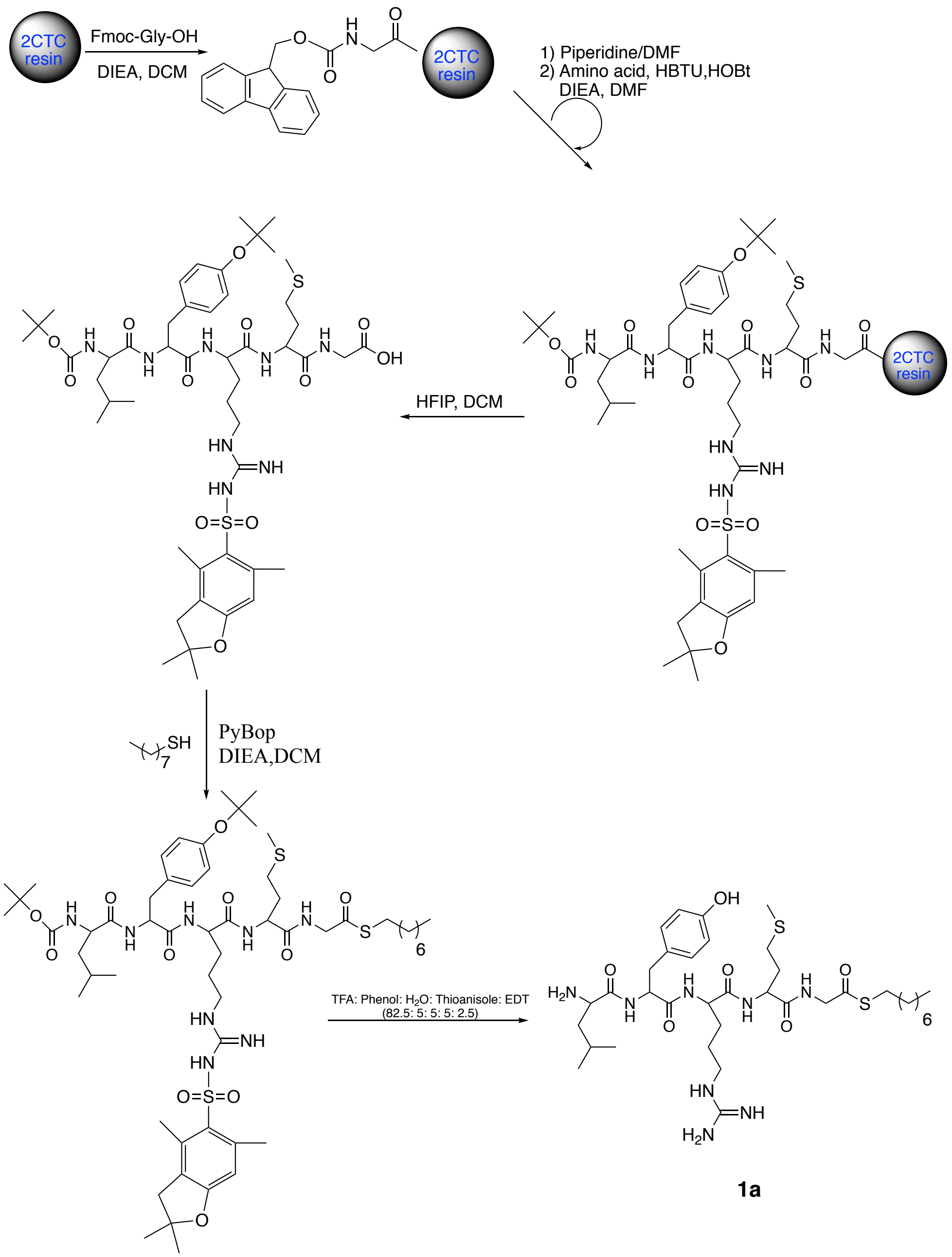

1a

Supplementary Figure 49. Synthesis of LYRMG-αCOSC8 (**1a**).

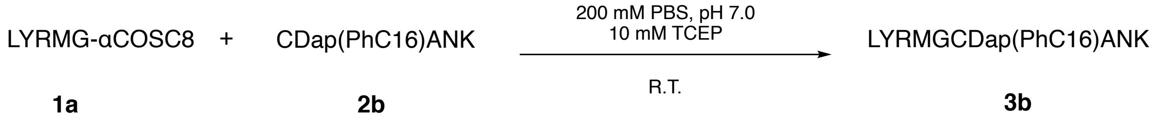

Supplementary Figure 50. Ligation reaction between peptide **1a** and peptide **2b**.

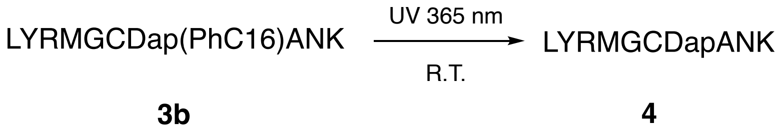

Supplementary Figure 51. Photouncaging reaction to generate LYRMGCDapANK (**4**).

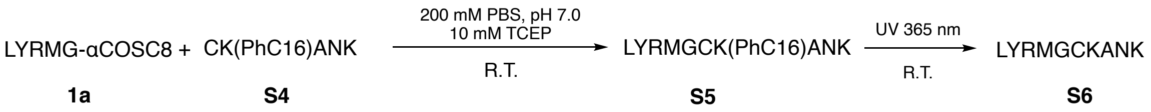

Supplementary Figure 52. Synthesis of LYRMGCKANK (**S6**).

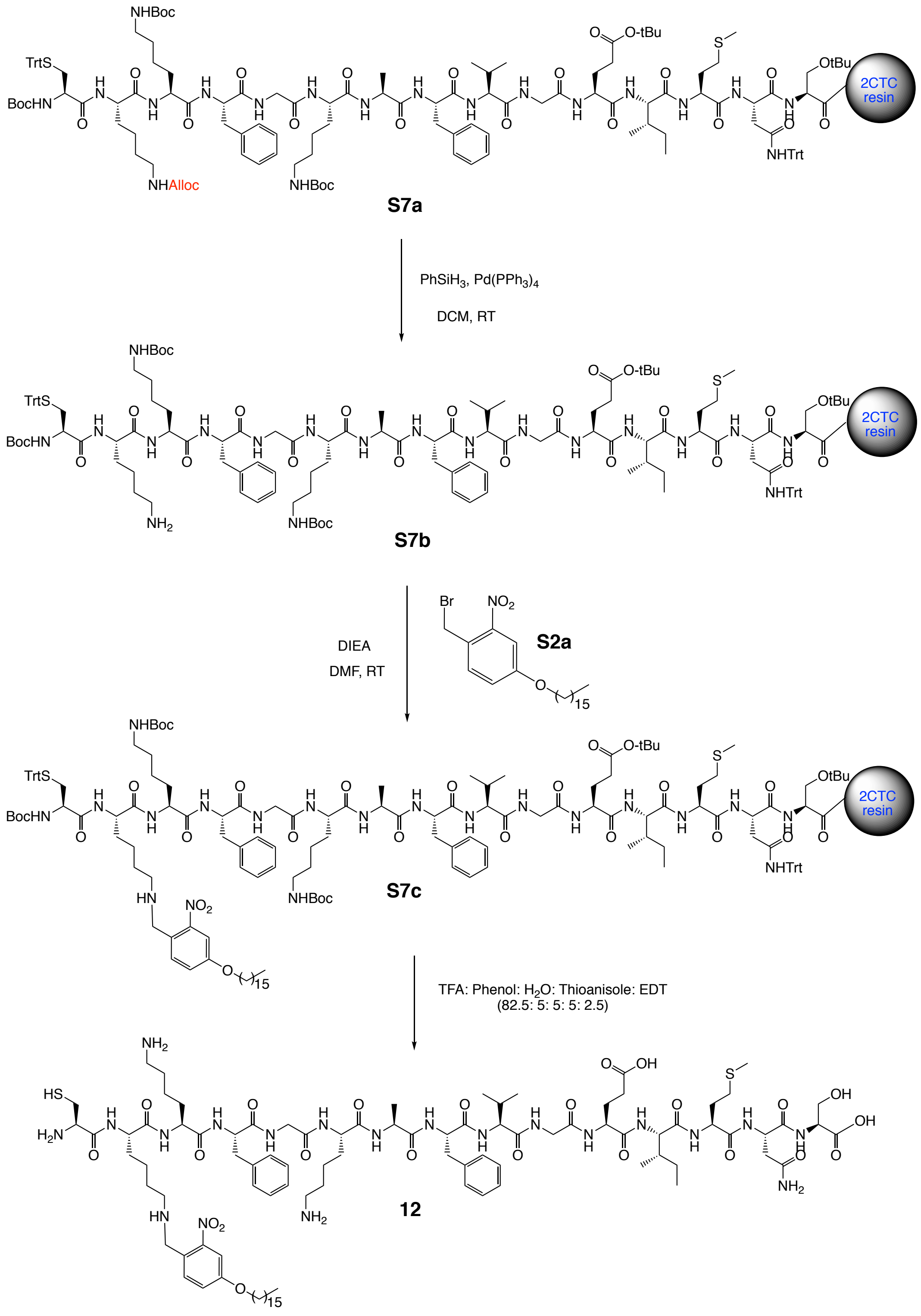

Supplementary Figure 53. Synthesis of CK(PhC16)KFGKAFVGEIMNS **(12)**.

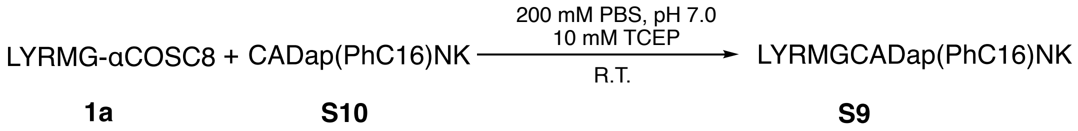

Supplementary Figure 54. Synthesis of LYRMGCADap(PhC16)NK (**S9**).

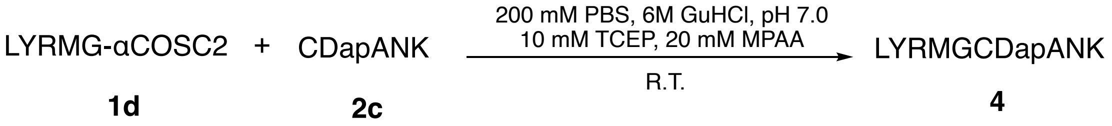

Supplementary Figure 55. Ligation reaction between peptide **1d** and peptide **2c**.

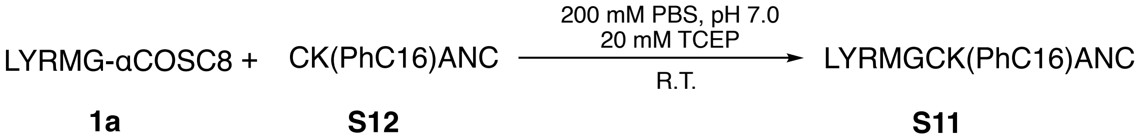

Supplementary Figure 56. Synthesis of LYRMGCK(PhC16)ANC (**S11**).

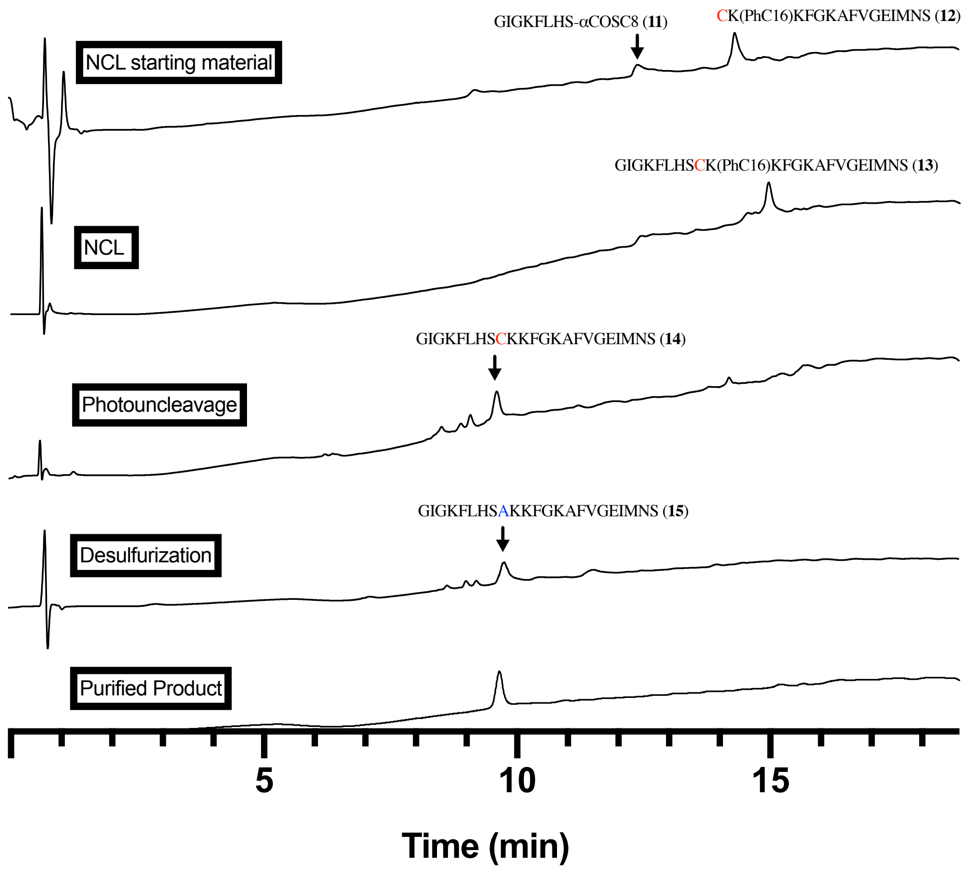

Supplementary Figure 57. Full spectra corresponding to the synthesis of Magainin 2 (**15**).

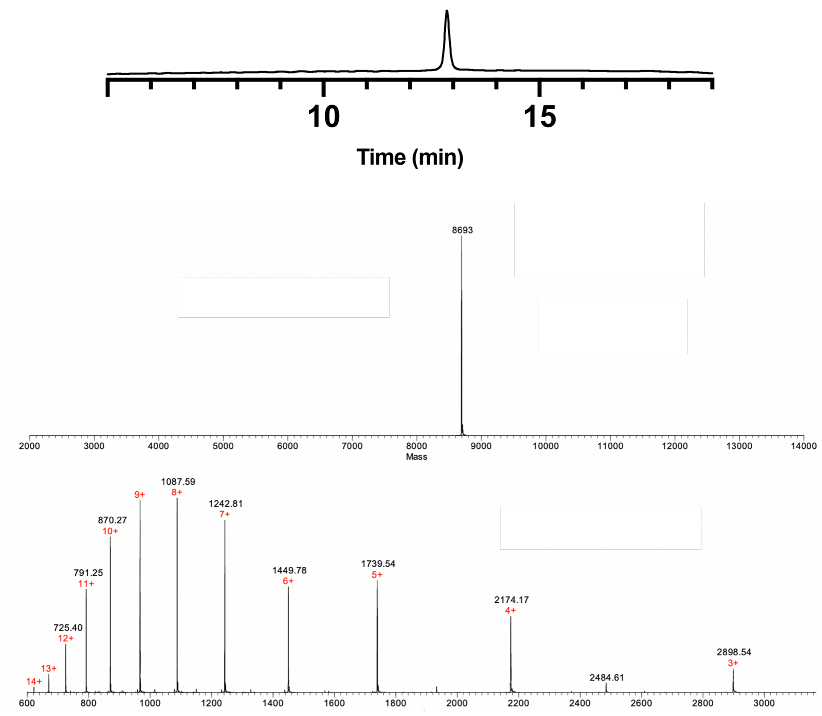

Supplementary Figure 58. HPLC trace and MS (ESI) of purified Ubi-αCOSC8 (**16**).

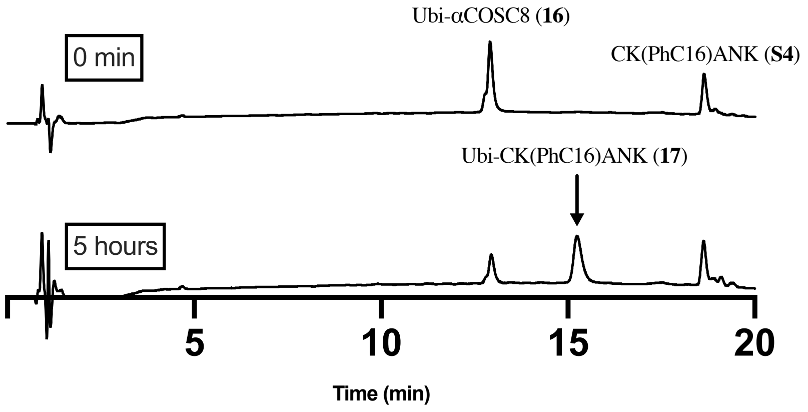

Supplementary Figure 59. Full spectra corresponding to the NCL-based derivatization of Ubiquitin.

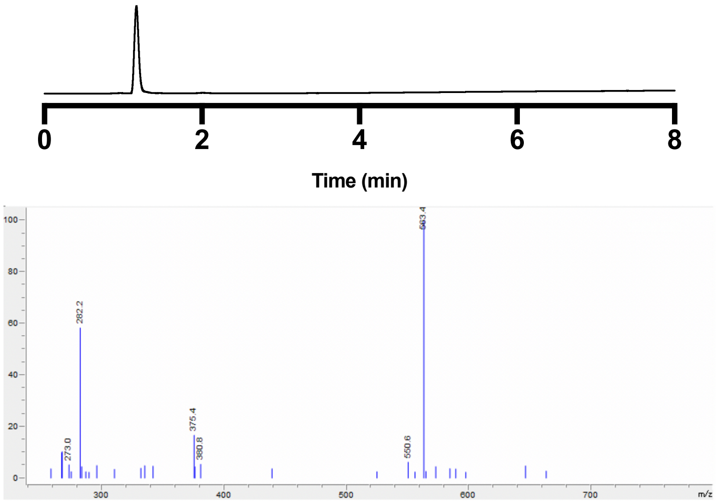

Supplementary Figure 60. HPLC trace and MS (ESI) of purified CKANK (**S13**).

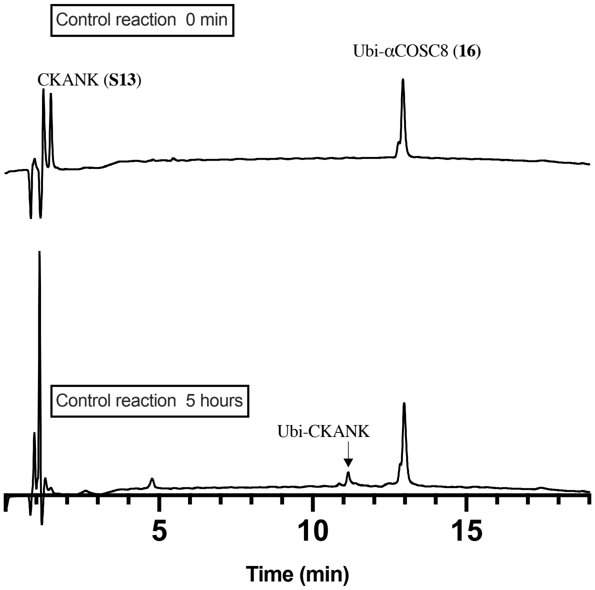

Supplementary Figure 61. Full spectra corresponding to the control reaction for the derivatization of Ubiquitin.

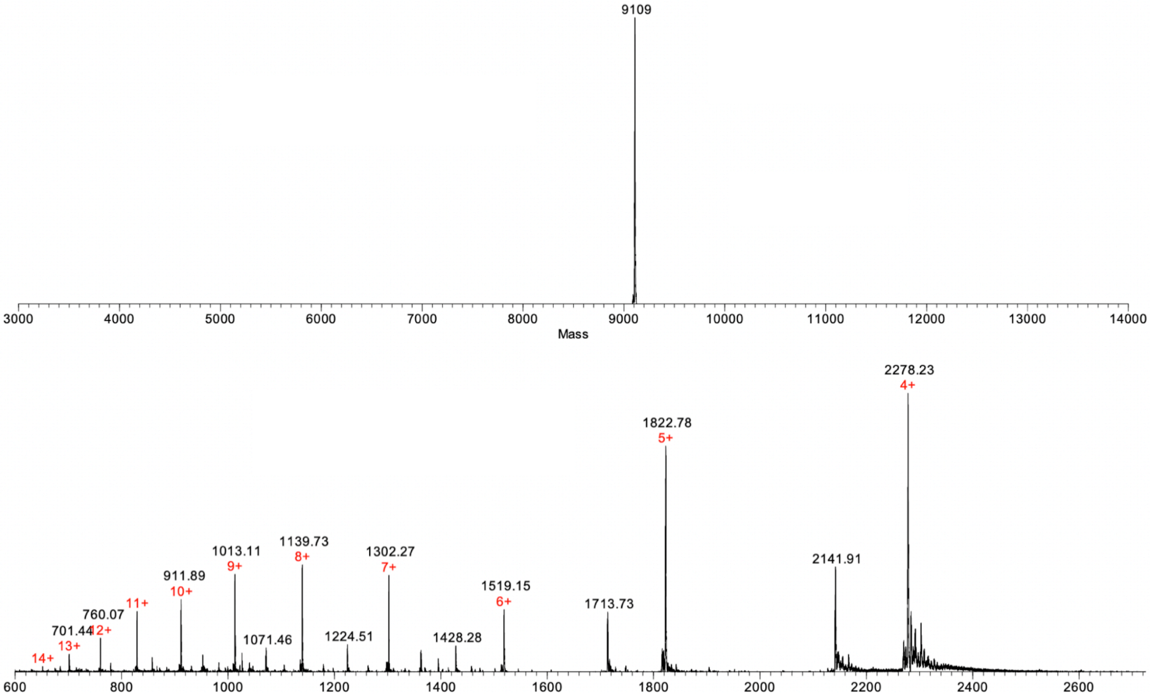

Supplementary Figure 62. MS (ESI) of Ubi-CKANK (**S14**).

Supplementary References

1. Dang, B., Kubota, T., Mandal, K., Bezanilla, F. & Kent, S. B. H. Native chemical ligation at Asx-Cys, Glx-Cys: chemical synthesis and high-resolution X-ray structure of ShK toxin by racemic protein crystallography. *J. Am. Chem. Soc.* **2013**, *135*, 11911-11919.
